# Transcriptional timing and noise of yeast cell cycle regulators—a single cell and single molecule approach

**DOI:** 10.1038/s41540-018-0053-4

**Published:** 2018-05-21

**Authors:** Aouefa Amoussouvi, Lotte Teufel, Matthias Reis, Martin Seeger, Julia Katharina Schlichting, Gabriele Schreiber, Andreas Herrmann, Edda Klipp

**Affiliations:** 1 Theoretical Biophysics, Institute of Biology, Humboldt-Universität zu Berlin, Berlin, Germany; 2 Molecular Biophysics, Institute of Biology, Humboldt-Universität zu Berlin, Berlin, Germany

## Abstract

Gene expression is a stochastic process and its appropriate regulation is critical for cell cycle progression. Cellular stress response necessitates expression reprogramming and cell cycle arrest. While previous studies are mostly based on bulk experiments influenced by synchronization effects or lack temporal distribution, time-resolved methods on single cells are needed to understand eukaryotic cell cycle in context of noisy gene expression and external perturbations. Using smFISH, microscopy and morphological markers, we monitored mRNA abundances over cell cycle phases and calculated transcriptional noise for *SIC1*, *CLN2*, and *CLB5*, the main G1/S transition regulators in budding yeast. We employed mathematical modeling for in silico synchronization and for derivation of time-courses from single cell data. This approach disclosed detailed quantitative insights into transcriptional regulation with and without stress, not available from bulk experiments before. First, besides the main peak in G1 we found an upshift of *CLN2* and *CLB5* expression in late mitosis. Second, all three genes showed basal expression throughout cell cycle enlightening that transcription is not divided in on and off but rather in high and low phases. Finally, exposing cells to osmotic stress revealed different periods of transcriptional inhibition for *CLN2* and *CLB5* and the impact of stress on cell cycle phase duration. Combining experimental and computational approaches allowed us to precisely assess cell cycle progression timing, as well as gene expression dynamics.

## Single-cell approach dissects noisy cell cycle transcription

Single-molecule time-resolved mRNA quantification revealed long-term regulation of transcription and cell cycle progression upon osmotic stress. A team led by Edda Klipp at Humboldt-Universität zu Berlin, Germany, combined smRNA-FISH microscopy, genetic and morphological markers and stochastic modeling to monitor mRNA dynamics and related noise levels throughout cell cycle for *SIC1, CLN2*, and *CLB5*, main G1/S regulators in budding yeast. All genes were present throughout cell cycle enlightening that transcription is not divided in on and off but rather in high and low phases. Osmotic stress induced distinct periods of inhibition for *CLN2* and *CLB5*, and long-term impact on cell cycle phase progression. This approach provided insights in transcription regulation unavailable with bulk experiments and chemical synchronization. Future research may address whether these patterns are general to other eukaryotic cell cycle regulators.

## Introduction

Correct gene expression regulation is crucial for cell cycle progression.^1^ Main regulators of the cell cycle are cyclins, cyclin dependent kinases (CDK) and CDK-inhibitors (CKI).^2^ Their functions and regulatory motifs are highly conserved among eukaryotes.^3,4^ Gene expression is frequently measured for cell cycle synchronized populations despite the facts that synchronization affects cell cycle progression heavily and that single cell behavior deviates from population behavior. Therefore, we aimed for a more precise analysis of transcriptional dynamics during the cell cycle.

For this work, three well-studied examples for cell cycle regulators in budding yeast were selected: Clb5, Cln2, and Sic1. The two cyclins Clb5 and Cln2 in complex with CDK1 control replication origin firing and bud formation, respectively, characterizing the exit from G1 and entrance into S phase.^5–7^ The CDK inhibitor Sic1 prevents premature G1/S transition, also called START, by inhibiting Clb5-CDK1 during G1 phase.^8^ At START Cln2 production, in turn, induces Sic1 hyperphosphorylation, ubiquitination, degradation and consequently the entrance into S phase.^9^ *CLN2* and *CLB5* belong to the G1 gene cluster and their mRNA levels peak in late G1 phase.^10,11^ *SIC1* transcription is mainly induced by two transcription factors, Swi5 in late mitosis and Ace2 in newborn daughter cells in early G1.^12–15^

Besides the precise timing of different processes of cell cycle progression under normal growth conditions, the selected genes are involved in stress response. Stress adaptation is critical, since its dysfunctions can lead to genomic instability.^16^ Exposure to high concentrations of osmolytes activates the stress MAP kinase Hog1, responsible for downregulation of *CLN2* and *CLB5* transcription and stabilization of Sic1 through phosphorylation, preventing its ubiquitination and consequently delays exit from G1.^17^ Furthermore, studies using synchronized cell populations showed that cells also arrest in G2^18,19^ and that the S phase is delayed and elongated.^16,20^ However, the immediate influence of osmotic stress on transcription in unsynchronized cells and the long-term response remain elusive.

Understanding the function of cellular regulatory networks under normal and perturbed conditions requires precise data as basis for the development of a consistent quantitative model of the dynamic behavior of these networks.^21,22^ Genome-wide assays on populations synchronized with α-factor (early G1), nocodazole (G2/M) or temperature-sensitive cdc15-2 mutant (G2/M) revealed the dynamics of genes controlling cell cycle,^23–27^ but these methods are known to perturb cell cycle regulation.^28–30^ Besides, synchrony within a population is usually not retained over the entire cell cycle, leading to a lack of precise information for later or short events in G2 and M phases. As progression of the synchronized population is relative to the time of release from the synchronizing agent, measured time-courses are challenging to link to specific cell cycle phases.

Established experimental techniques like RNA sequencing or quantitative PCR provide mostly relative mRNA numbers on the population level with extremely high variation of low abundant transcripts.^31^ Absolute enumeration of mRNA molecules in single cells by smFISH confirmed the low transcript numbers found in the genome-wide assays, and showed transcriptional variability among cells in a population, which is considered as transcriptional noise.^32–40^ Such single cell microscopy methods on fixed cells usually lack timing information on cell cycle dynamics. Consequently, time-resolved monitoring of absolute changes of mRNA numbers for cell cycle regulating genes is still missing to understand and model the transcriptional network, and its robustness against external stimuli (perturbations). In order to assess critical decisions during yeast cell cycle and to characterize the impact of noise in the light of small molecule numbers, a precise quantification of the temporal behavior is essential.

**Fig. 1:**
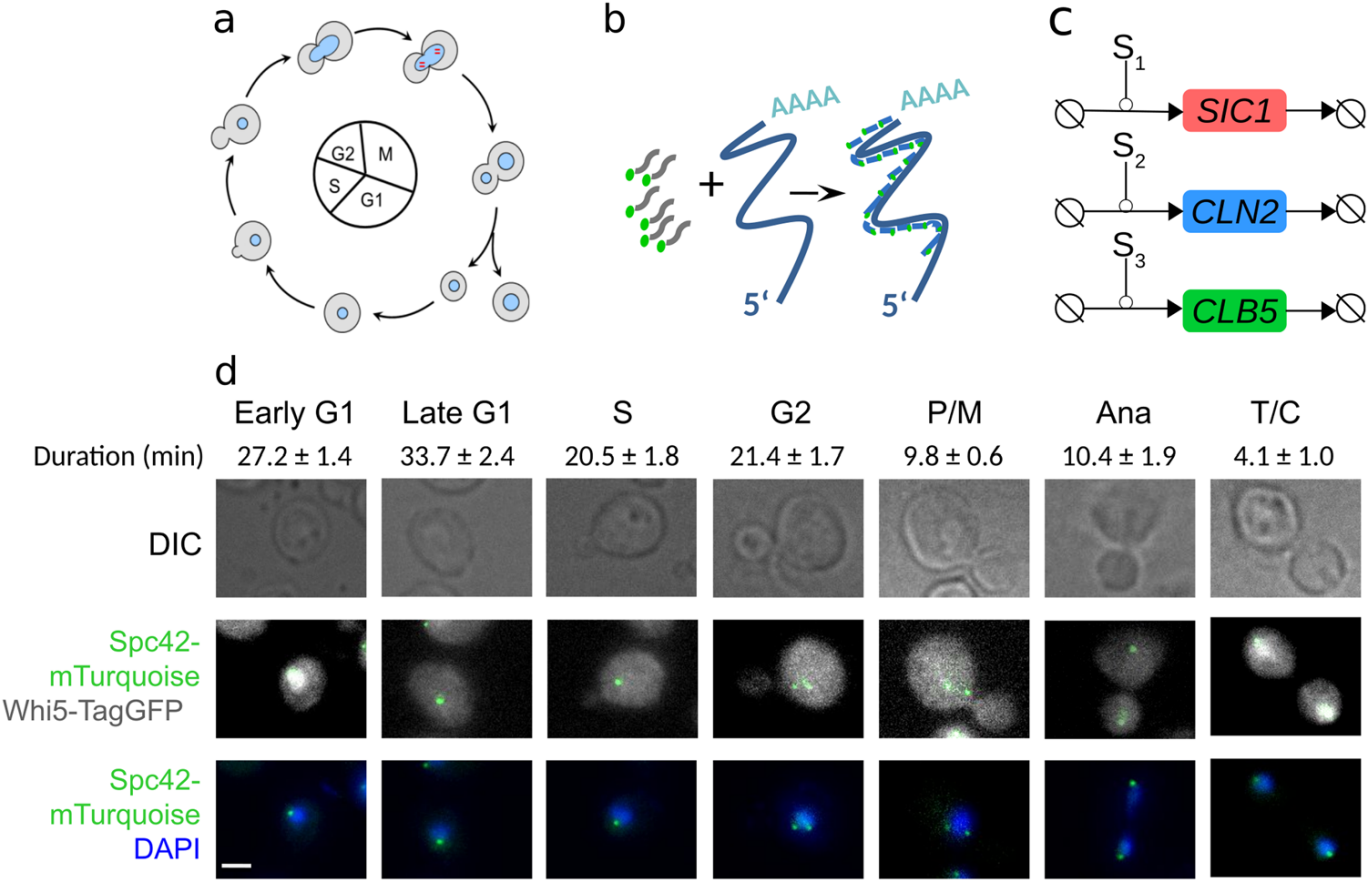
Methods and strategy. **a** Schematic cell cycle progression of *S. cerevisiae*. **b** Principle of smFISH method. A set of fluorescently labeled DNA probes hybridizes with the target mRNA sequence. Single mRNA molecules can be detected by fluorescence microscopy. **c** *SIC1*, *CLN2*, and *CLB5* transcription and degradation. S_1_, S_2_, and S_3_ denote signals for transcription upregulation in the models. **d** Morphological and genetic cell cycle progression markers allow to discriminate seven different cell cycle phases (early G1, late G1, S, G2, P/M, Ana, T/C). Markers used were the presence and size of a bud (first row), the number and orientation of the spindle pole bodies (green spots in second and third rows), the localization of Whi5 (white in second row) and the shape and number of nuclei (blue in third row). Scale bar represents 2 µm. The duration (mean +/− SEM) of the phases has been determined according to the number of cells found in each phase (*n* > 2600 cells, see Biological Replicates in Supplementary Information)

Here, we combined quantitative in vivo single molecule RNA-Fluorescence in situ hybridization (smFISH) experiments, in silico synchronization and stochastic modeling to precisely assess cell cycle progression timing and gene expression dynamics and variability. The strategy is illustrated in Fig. 1. We used asynchronous cell populations to avoid adverse influences caused by cell cycle synchronization methods. Instead, we used genetic and morphological markers to assign cells to specific cell cycle phases (Fig. 1d), counted mRNA numbers per cell in each phase and used the resulting distributions of mRNAs per phase to fit a time-resolved mathematical model, which we called in silico synchronization.

In detail, we quantified absolute transcript levels of *SIC1*, *CLN2*, and *CLB5* in single cells and noise levels within a population in different cell cycle phases. We found that transcription of the three genes is never turned off, instead an enduring basal level throughout the entire cell cycle was detected. The basal level for the CKI *SIC1* was higher than for the two cyclins. Each gene exhibited distinct expression pattern for periods of high transcription: *SIC1* had a very sharp transcription peak in M phase with an almost instant decay. Both cyclins had their main transcription peak in late G1. *CLN2* exhibited a longer period of transcriptional activity with slower decay than *SIC1* and *CLB5* showed a longer period of moderate transcription. Additionally, we observed an increase of *CLN2* and *CLB5* transcription in mitosis. Using a simple stochastic model for transcription and mRNA decay, we derived respective rate constants, as well as timing of periods of high and low expression and their noise levels.

For the case of osmotic stress, our model determined two distinct periods of transcriptional repression for the two cyclins, indicating different osmoregulation mechanisms, which could be related to a loss of synchrony between the timing of DNA synthesis and budding observed in our study. Moreover, studying osmostress-induced long-term changes we elucidated a shift of gene expression to later cell cycle phases.

## Results

### Transcript distributions for *SIC1*, *CLN2*, and *CLB5* in an asynchronous population

We measured absolute transcript numbers of *SIC1*, *CLN2*, and *CLB5* in single budding yeast cells of an asynchronous population with smFISH. We normalized the intensity of each detected mRNA fluorescent spots by the median fluorescence intensity of the spot population (see Supplementary Information Material and Methods). 96, 92, and 88% of the spots for *SIC1*, *CLB5*, and *CLN2*, respectively, contained one single mRNA molecule. Nuclear spots containing at least three mRNA molecules were defined as transcription start sites (TS).^41^

**Fig. 2:**
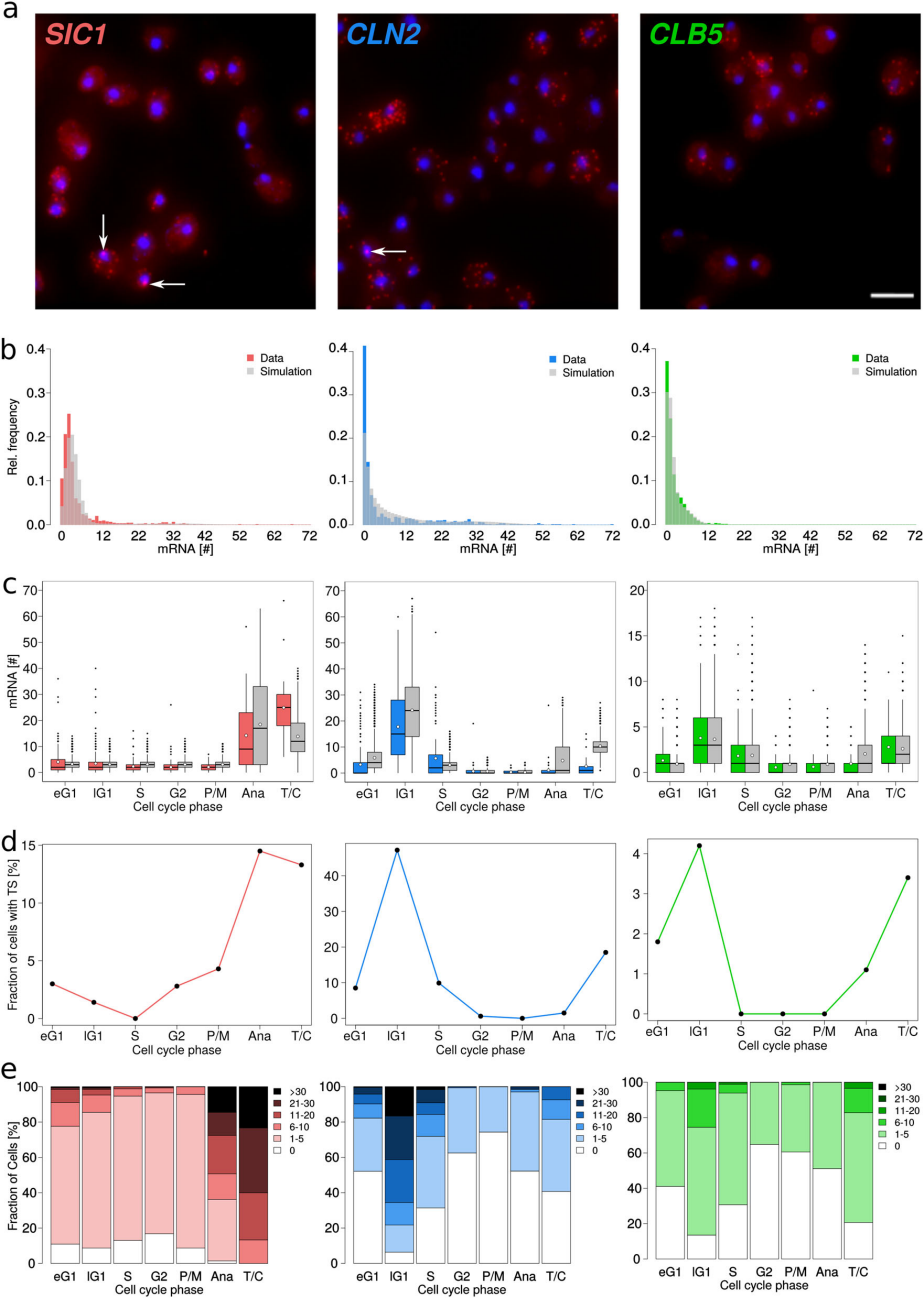
Images and quantitative analysis of single-cell transcription for *SIC1*, *CLN2*, and *CLB5* under optimal growth conditions in an asynchronous cell population. **a** Detection at single molecule resolution of endogenous *SIC1*, *CLN2*, and *CLB5* transcriptional expressions with smFISH (red dots: mRNA molecules; blue: DAPI-stained nucleus). White arrows point towards transcription sites. Scale bar represents 5 μm. **b** Experimental and simulated distributions of mRNA number per cell for *SIC1*, *CLN2*, and *CLB5* for the entire population (*n* > 900 cells). The overall averages are 4.53, 6.46, and 1.93 and medians are 2, 1, and 1 mRNAs/cell for *SIC1*, *CLN2*, and *CLB5*, respectively. **c** Measured and simulated mRNA distributions for each cell cycle phase. Lower and upper quartiles form boundaries of the boxes. Lines and white dots insight the boxes represent medians and mean values, respectively. Whiskers are given by 1.5 × IQR (interquartile range). Data points outside of the IQR define outliers. For better overview early G1 and late G1 are abbreviated to eG1 and lG1, respectively. In **b** and **c** experimental and simulated data are represented by colored and gray bars or boxes, respectively. **d** Fraction of cells with transcription sites (TS) in each cell cycle phase. Lines between data points are for visualization only. **e** mRNA distributions for each cell cycle phase grouped as follows: 0, 1–5, 6–11, 12–20, 21–30, >30 number of mRNA per cell (*n* > 900 cells see Supplementary Table S1 for data)

Microscopic images and frequency distributions of mRNA numbers per cell showed different patterns for the three genes (Fig. 2a, b and Supplementary Table S1) with averages of 4.53, 6.46, and 1.93 and medians of 2, 1, and 1 mRNAs/cell for *SIC1*, *CLN2*, and *CLB5*, respectively. These values were in the same order of magnitude as other smFISH studies on budding yeast^35,41,42^ (Supplementary Fig. S1). Ninety percent of the cells contained at least one *SIC1* mRNA indicating that the transcription is not turned off completely, whereas *CLN2* and *CLB5* mRNAs were absent from about 40% of the population (Fig. 2b). *SIC1* and *CLN2* had long distribution tails (Fig. 2b).

### Markers for cell cycle phase

To investigate changes of mRNA abundances over cell cycle we used an asynchronous cell culture in combination with genetic and morphological markers to assign each cell to a cell cycle phase. These are: presence and size of a bud, morphology of the DAPI-stained nucleus, number and localization of spindle pole bodies visualized by mTurquoise-labeled Spc42, and localization of TagGFP-labeled Whi5^41^ (Supplementary Information Material and Methods). Whi5 is recruited to the nucleus between late M and early G1 and is located in the cytoplasm during the rest of the cell cycle.^43^ We distinguished seven phases, i.e., early G1, late G1, S, G2, prometa-/metaphase (P/M), anaphase (Ana) and telophase/cytokinesis (T/C) (Fig. 1d). Tagging of Whi5 and Spc42 did not alter cell cycle progression as we obtained similar growth rates and mRNA distributions of *SIC1*, *CLN2*, and *CLB5* for wild type and the strain containing Whi5-TagGFP and Spc42-mTurquoise (Supplementary Fig. S2). The doubling time was 129 min in YPD at 30 °C (124 min for wild type). Phase durations were determined as being proportional to the fraction of cells in each phase^41^ (Fig. 1d). For determination of cell cycle phases, we used more than 2600 cells.

### *SIC1* mRNA is present throughout cell cycle

*SIC1* transcript level was minimal between G1 and early mitosis, i.e., P/M, and increased in anaphase from its basal level of about 2 mRNAs/cell to a maximum of about 25 mRNAs/cell in T/C (Fig. 2c). It dropped to about 4 mRNAs/cell when cells entered into next early G1 and further decreased until S phase. The *SIC1* mRNA transcription maximum in T/C, i.e., late mitosis, is consistent with previous studies.^12–14^ The percentage of cells containing active transcription sites reached a maximum in Ana and T/C phases (Fig. 2d, Supplementary Fig. S3). To conclude, basal *SIC1* presence is robust over the cell cycle. Indeed, in S, G2, P/M, the phases of lowest *SIC1* expression, more than 80% of the cells contained at least one *SIC1* mRNA (Fig. 2e).

### *CLN2* and *CLB5* transcription shows enduring basal levels and rises in late mitosis

As expected for genes of the G1 cluster, *CLN2*, and *CLB5* showed transcription maxima in late G1 (Fig. 2c). We observed an unexpected transcriptional activity during late mitosis with 60% and 80% of cells containing at least one mRNA of *CLN2* and *CLB5*, respectively, in T/C (Fig. 2e). Since only about 3% of the cells were in T/C, we would need higher cell numbers to statically characterize this phenomenon. Transcript levels of *CLN2* and *CLB5* were low in early G1 and increased to a maximum of about 18 and 4 mRNAs/cell, respectively, in late G1. During late G1, 94% and 86% of cells contained at least one transcript of *CLN2* or *CLB5*, respectively (Fig. 2e). After G1/S transition transcript levels dropped. From G2 until mitosis, *CLN2* and *CLB5* levels were minimal, however, about 30% of cells contained at least one mRNA. The dynamics of transcription sites displayed maxima in late G1 and T/C (Fig. 2d, Supplementary Fig. S3).

### Computational model for mRNA dynamics—rationalizing experimental data

To understand mRNA dynamics based on static, but time-resolved, single cell smFISH data we stochastically modeled transcription and degradation for each species. In the model, signals S_1_ to S_3_ indicate the upregulation of transcription for *SIC1* (S_1_), *CLN2* (S_2_), and *CLB5* (S_3_) (Fig. 1c). We assumed equal degradation rate constants (_*pi*_) and different transcription (*k*_*ih*_ or *k*_*il*_) rate constants in periods of either high (*h*) or low (*l*) expression (Table 1). Periods of high expression are defined by transcription start time ($t_{S_{i,0}}$) and transcription end time ($t_{S_{i,e}}$). We assumed a second period of high transcription for *CLN2* and *CLB5*, labeled as *first* and *sec*. The duration of one cell cycle is set to *t* = 129 min.

**Table 1:** Reactions and parameters

Process	Reaction scheme	Rate	Parameter values [min^-1^]	Transcription times [min]
*SIC1* transcription	→*SIC*1	*k*_1_⋅*S*_1_	*k*_1*h*_ = 22.15	$t_{S_{1,0}} = 118.09$
			*k*_1*l*_ = 1.72	$t_{S_{1,e}} = 124.05$
*SIC1* degradation	*SIC*1→	*p*_1_⋅*SIC*1	*p*_1_ = 0.56	
*CLN2* transcription	→*CLN*2	*k*_3_⋅*S*_2_	*k*_3*h*_ = 4.4	$t_{S_{2,0,first}} = 20.57$
			*k*_3*l*_ = 0.02	$t_{S_{2,e,first}} = 39.83$
				$$t_{S_{2,0,sec}} = 119.01$$
				$$t_{S_{2,e,sec}} = 124.05$$
*CLN2* degradation	*CLN*2→	*p*_3_⋅*CLN*2	*p*_3_ = 0.09	
*CLB5* transcription	→*CLB*5	*k*_5_⋅*S*_3_	*k*_5*h*_ = 3.05	$t_{S_{3,0,first}} = 40.16$
			*k*_5*l*_ = 0.46	$t_{S_{3,e,first}} = 62.59$
				$$t_{S_{3,0,sec}} = 120.26$$
				$$t_{S_{3,e,sec}} = 124.35$$
*CLB5* degradation	*CLB*5→	*p*_5_⋅*CLB*5	*p*_5_ = 0.51	

This table lists all elementary processes and the respective reaction schemes as shown in Fig. 1. All reactions follow mass action kinetics. The rate expressions are provided, as well as the respective parameter values. The last column provides the start and end times for high expression of the respective genes

Because the mRNA numbers were measured as distributions per cell cycle phase, a specific approach in parameter estimation was required. Since the mRNAs for each gene evolved statistically independently, we could estimate parameter values and transcription start and end times separately. We used the analytical solution of the chemical master equation for monomolecular reactions with Poisson initial conditions. We obtained the best results with a local optimizer falling into the class of “line search” algorithms (see Supplementary Information, Parameter estimation). Parameter values and transcription start and end times of our stochastic model were fitted to best reproduce the time-resolved frequency distributions of *SIC1*, *CLN2*, and *CLB5* mRNAs (Fig. 2b,c, Supplementary Table S1 and Supplementary Fig. S4).

Transcription dynamics could only be fitted by assuming low, but non-zero, transcription outside the main transcription periods with relative promoter activities of 7.7, 0.4, and 15% for *SIC1*, *CLN2*, and *CLB5*, respectively. According to the Akaike criterion (AIC), the fits improved when allowing for two periods of high promoter activity for *CLN2* and *CLB5*.

**Fig. 3:**
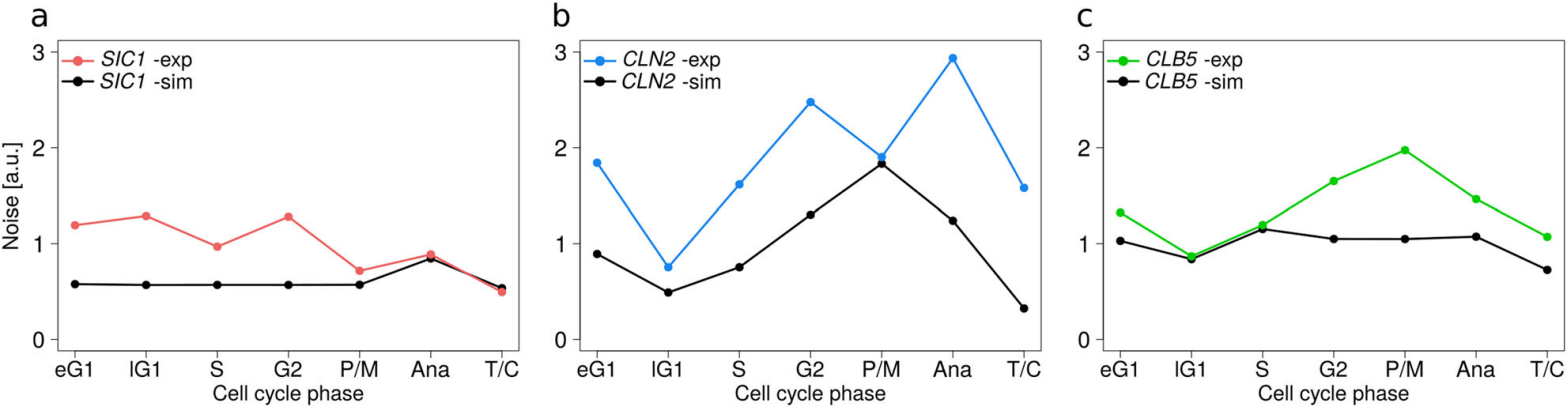
Experimental and simulated noise of mRNA numbers in cell cycle phases. Noise is calculated as ratio of standard deviation to mean. Noise of mRNA numbers for *SIC1* (**a**), *CLN2* (**b**), and *CLB5* (**c**) has been determined either from the smFISH experimental data (*n* > 900 cells per genes. see Supplementary Table S1 for data) or from the stochastic simulations (*n* = 2000 simulated cells). Lines between data points are for visualization only

**Fig. 4:**
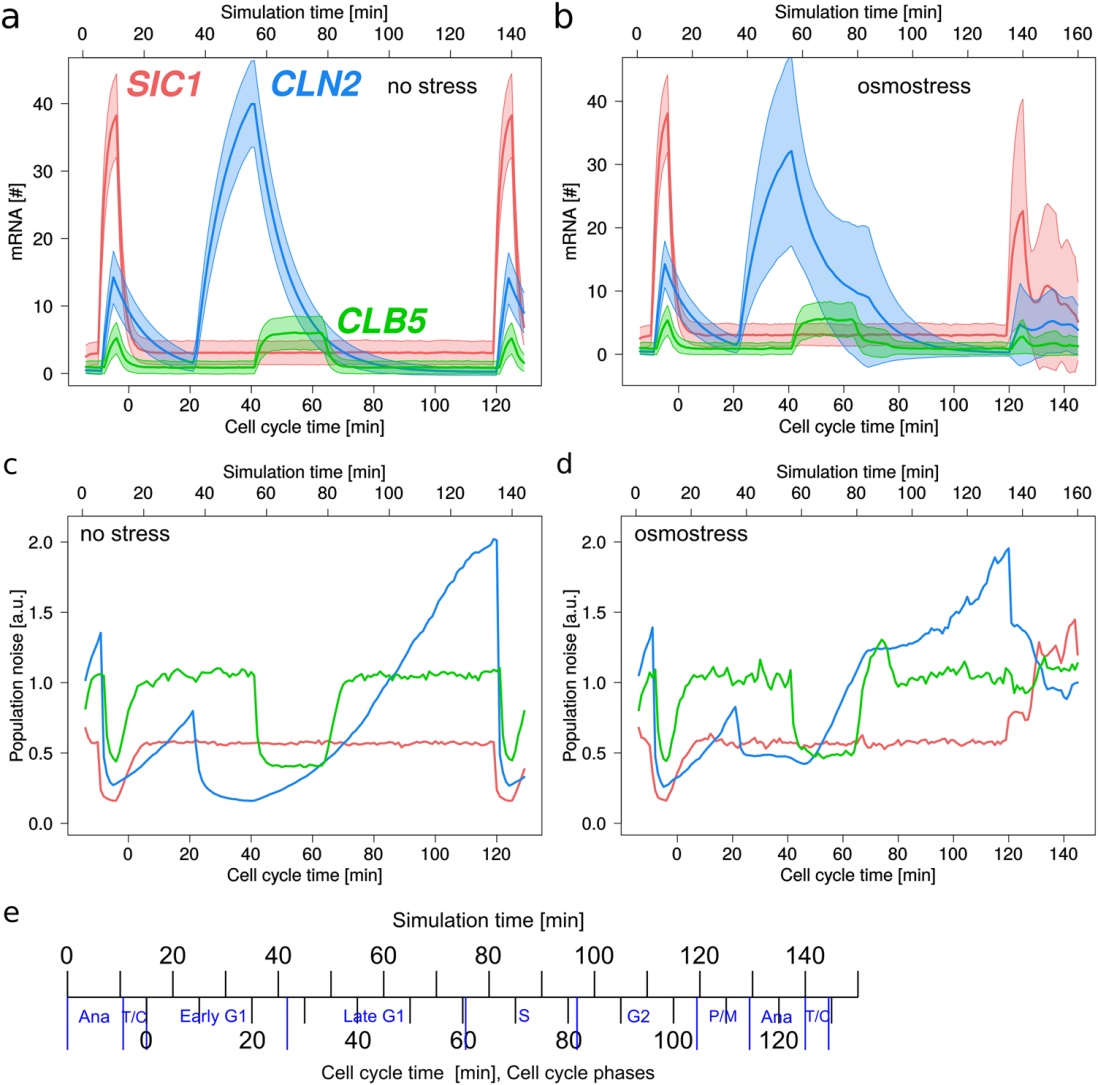
Simulated time-courses of transcript numbers and molecular noise during cell cycle for *SIC1*, *CLN2* and *CLB5* under optimal conditions and under osmostress. Simulations were performed with the Gillespie algorithm using the equations and parameter values provided in Table 1. 2000 cells were simulated from anaphase until T/C phase of the next cell cycle, leading to a shift of 15 min between simulation time and cell cycle time. Transcript levels increase during periods of active signals S_1_, S_2_, or S_3_ (see Fig. 1b). **a,** **b** Time-courses of mean values (thick lines) and standard deviations (shaded areas around thick lines) of mRNA under optimal conditions and under osmostress, respectively. **c**, **b** Time-courses of noise measured over the whole population, i.e., standard deviation divided by mean of all 2000 cells at each time point. **e** Relation between cell cycle and simulation time

We analyzed the noise for experimental mRNA abundances and for simulated mRNAs using the coefficient of variation (CV), i.e., ratio of standard deviation to mean (Fig. 3, Supplementary Fig. S13). Except of in late G1, *SIC1* transcript population had the lowest experimental CV values and, therefore, the least degree of noise. Time-course simulations for mRNA numbers (Fig. 4a), noise over time (Fig. 4b), and noise per cell during one cell cycle period (see Supplementary Fig. S5) reproduced the experimental data (Fig. 2b,c, Fig. 3, Supplementary Fig. S4). Noise calculated in periods of either high or low expression both from the parameter values and based on the simulated time-courses (Fig. 3, Supplementary Fig. S5) revealed lower noise in high expression phases than in low expression phases.

### Osmostress induces changes in the timing of cell cycle phases

To test the robustness of G1/S transition with respect to perturbations, we exposed yeast cells to hyperosmolarity. Dependent on the cell cycle phase, cell cycle progression is delayed to allow stress adaptation.

**Fig. 5:**
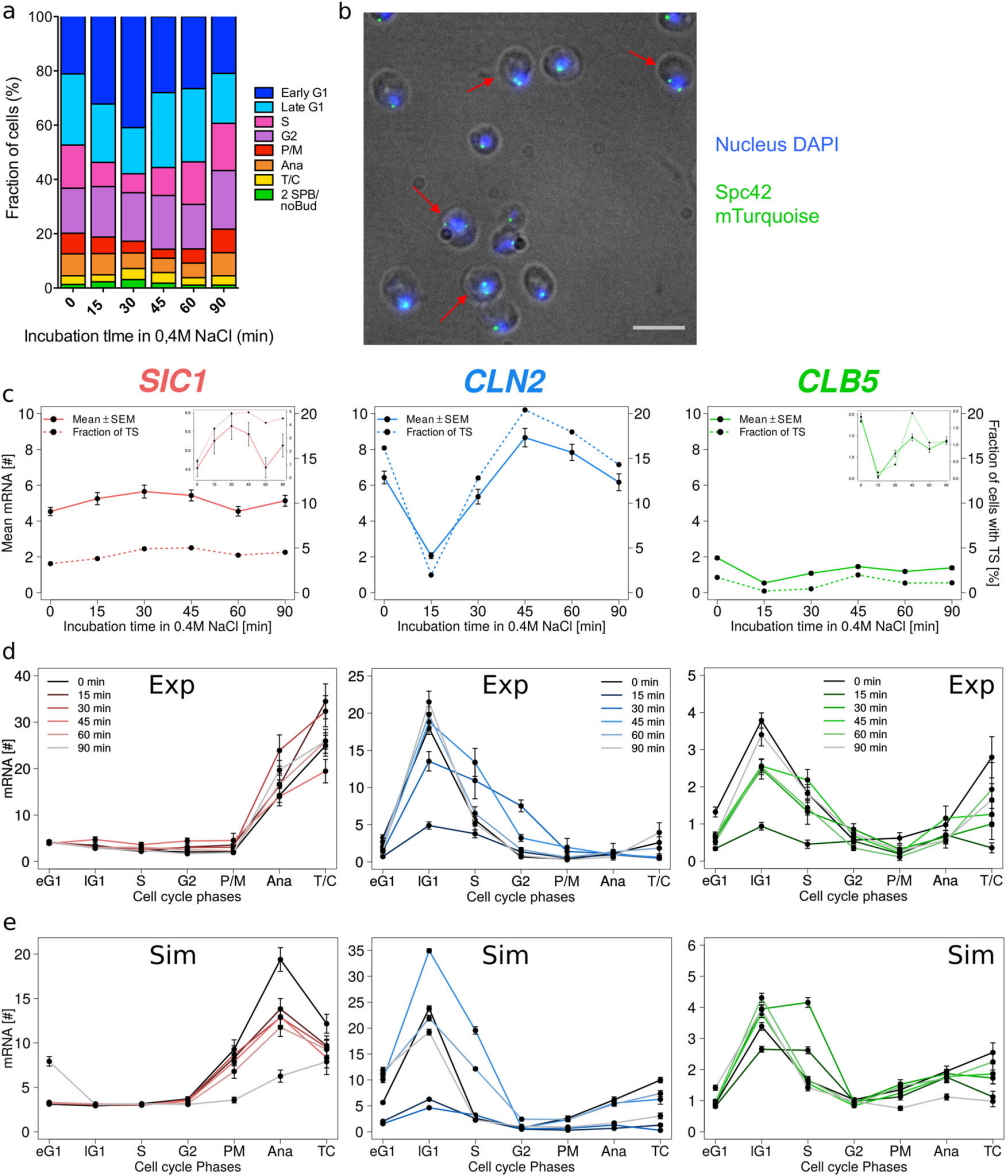
Medium osmotic stress affects cell cycle progression and transcription. **a** Fraction of cells in each cell cycle phases before and during exposure to osmotic stress. Numerical values in Supplementary Fig. S6. The cells were assigned to each phase using the markers previously described in Fig. 1 (*n* > 1500 cells per time point). **b** Fluorescence microscopy images depicting a fraction of cells that experience a loss of synchrony between DNA replication and budding under osmotic stress. Red arrows point to cells containing two separated spindle pole bodies without bud. The image is an overlay of bright field image, DAPI-stained nucleus (in blue) and mTurquoise tagged Spc42 part of the spindle pole body (in green). Scale bar represents 5 μm. (**c**) and (**d**) *n* > 400 cells per genes and time points. Raw data in Supplementary Table S1 and Biological Replicates in Supplementary Information. **c** Changes in mRNA levels shown as mean ± SEM (dots and full lines) and transcription activity represented by percentage of cells with a transcription site (TS) (dots and dotted lines). Plot insets represent a zoom in. **d** Influence of osmostress on timing of mRNA abundances for *SIC1, CLN2*, and *CLB5* during the cell cycle. Data shown are mean ± SEM. Darkest curves correspond to the shortest incubations in hyperosmotic medium. (**e**) Simulated changes of timing of mRNA abundances for *SIC1, CLB5*, and *CLN2* during the cell cycle progression upon osmotic stress (*n* = 2000 cells). Data shown are mean ± SEM. Details of (D) and (E) depicted in Supplementary Fig. S12. In **a**, **c**, **d,** and **e** 0 min is equivalent to no stress conditions. Lines between data points in **c**, **d,** and **e** are for visualization only

To monitor stress response, we applied 0.4 M NaCl to a cell culture for 0, 15, 30, 45, 60, and 90 min and assigned each cell to a cell cycle phase (Fig. 5a and Supplementary Fig. S6). While cell cycle progression without stress is fully characterized, the cell cycle phase lengths upon stress reaction are affected to a so far unknown degree and, hence the sensitivity during different cell cycle phases to stress and the full cell cycle length remained undetermined. We found that the fraction of cells in each cell cycle phase, and hence the length of that phase, was affected differently and even after 90 min under stress conditions the distributions of cells in each phase had not totally recovered to the non-stress situation (Fig. 5a, Supplement Fig. 6). Cells accumulated in early G1 until 30 min and in G2 until 45 min of stress, respectively. The fractions of cells in both phases increased from 21.1 to 41.0% for early G1 and from 16.6 to 19.8% for G2. Simultaneously, the fractions of cells in late G1, S, and in mitosis (P/M, Ana, and T/C) decreased. Interestingly, the decrease of the mitosis fraction lasted longer than the one of late G1 and S phase fractions. Indeed, we observed that recovery of the mitosis fraction started after 60 min whereas those of late G1 and S fractions already after 30 min. The measured results are in agreement with our previous simulations obtained from modeling a cell population under osmostress.^44^ Note, that since cells were not synchronized, the phase at which cells have been hit by stress could not be determined, especially after longer times of stress.

### Osmostress induces a temporary inhibition of *CLN2* and *CLB5* and an elongation of *CLN2* transcription to later cell cycle phases

Upon osmostress, *SIC1* mRNA levels and transcriptional dynamics were the least affected of the three genes (Fig. 5c, d and Supplementary Figs. S7 and S12A). *SIC1* mRNA maximum was detected at the end of mitosis in T/C (Fig. 5d) and its basal level stayed robust from early G1 until the beginning of mitosis in P/M. The mean *SIC1* level of the asynchronous population slightly increased within the first 30 min upon stress (Fig. 5c) due to the increase of *SIC1* in T/C and anaphase at 15 and 30 min stress exposure.

Contrarily, *CLN2* and *CLB5* mRNA levels declined within the first 15 min upon osmostress and showed distinct recovery patterns (Fig. 5c and d, Supplement Fig. S7). Within the first 15 min, *CLN2* and *CLB5* levels dropped from 6.4 to 2.0 and from 1.9 to 0.5 mRNA per cell, respectively, and later increased from 15 to 45 min. From 60 until 90 min of stress, *CLN2* mRNA levels recovered to unstressed transcription level, while *CLB5* levels at 90 min were still lower than before stress indicating only partial recovery within the cell population (Fig. 5c). The fraction of cells with transcription sites followed the behavior of their respective cyclin mRNA levels (Fig. 5c).

*CLN2* and *CLB5* transcriptional timing was also perturbed regarding individual cell cycle phases (Fig. 5d, Supplementary Figs. S7 and S12A). After 15 min of stress, the transcription of both cyclins was reduced. Both cyclin maxima in late G1 reappeared after 30 min and after 90 min the *CLN2* peak was higher than before stress while the *CLB5* maximum was not totally recovered, yet (Fig. 5d, Supplementary Fig. S7b, c). Interestingly, we observed a broadening of the *CLN2* mRNA peak in late G1 into G2 and P/M phase after 30 and 45 min of osmostress, respectively (Fig. 5d, Supplementary Fig. S7b). *CLB5* showed an extension of its transcription peak in late G1 into G2 at 45 min (Fig. 5d, Supplementary Fig. S7c).

### Partial loss of synchrony between DNA replication and bud morphogenesis under osmostress

Under normal growth conditions, budding and DNA duplication occur synchronously and are hallmarks of S phase entrance.^5–7^ Under hyperosmotic conditions, we observed a small fraction of cells (1.3% of the population under normal growth conditions, 3.1% after 30 min of osmostress, and 1.1% after 90 min) with two spindle pole bodies but without bud (Fig. 5a, green box, Fig. 5b, Supplementary Fig. S6), indicating loss of synchrony between bud morphogenesis and DNA duplication. Long-term observations of living cells over 5 h under salt stress revealed that the respective cells did not show any division (data not shown).

### Computational modeling of mRNA levels under osmotic stress

To simulate osmotic stress response of G1/S main regulators, we assumed that high-level transcription of *CLN2* and *CLB5* (mainly during late G1) was interrupted during the period of osmotic adaptation and resumed afterwards. Depending on the time point when osmotic stress hits the cell, the inhibitory effect on *CLN2* and *CLB5* transcription, as well as the delay of following cell cycle phases is different (for case-by-case analysis see Supplementary Fig. S8). Since all other model parameters were fitted already for the non-stress scenario, the remaining parameters to fit were the periods *t*_d_ of transcriptional repression. Employing the experimental single-cell mRNA distributions and means, we obtained values of *t*_d,*CLN2*_ = 28 min for *CLN2* and *t*_d,*CLB5*_ = 12 min for *CLB5*. To determine these values, we tested 676 combinations of *t*_d,*CLN2*_ and *t*_d,*CLB5*_ ranging from 5 to 30 minutes for each gene. We run 200 simulations for 200 random stress time points for each combination and compared the simulated distributions 0, 15, 30, 45, 60 and 90 minutes after stress with our experimental data. The best combination of *t*_d,*CLN2*_ and *t*_d,*CLB5*_ minimized the total squared distance between simulated and experimental mRNA distributions during individual cell cycle phases and at different time points upon stress (Supplementary Figs. S9-S11). Figure 4c, d show resulting time-courses for mRNA numbers and noise levels over time. As expected, the time-course of *SIC1* and its related noise were only slightly affected by osmotic stress (Fig. 4c, d Supplementary Fig. S13). Simulated *SIC1* levels stayed low from early G1 to P/M and displayed high expression in Ana and T/C (Fig. 5d, e, Supplement Fig. S12). Both *CLN2* and *CLB5* showed higher noise levels compared to no stress condition in late G1 and S phases (Fig. 4c, d). Both cyclins also experienced severe downregulation at 15 min of stress, especially for cells being in late G1 phase (Fig. 5d, e, Supplement Fig. S12). During recovery, cells being in S phase had higher *CLN2* and *CLB5* mRNA numbers than unstressed cells (Fig. 5d, e, Supplement Fig. S12).

## Discussion

### Stochastic modeling simulates time-courses of mRNA and noise from static single cell mRNA microscopy data

We applied smFISH to *SIC1*, *CLN2*, and *CLB5*, i.e., regulators of G1/S transition in yeast, and used genetic and morphological markers to assign cells to cell cycle phases and thereby obtained absolute and cell cycle phase-resolved mRNA numbers in single cells. Purposely, we avoided chemical or physical synchronization treatments. Computational modeling based on our experimental data enabled to estimate transcription kinetics parameters, to simulate time-courses of mRNA numbers and their noise, and to rationalize transcriptional responses and cell cycle timing upon external stress.

In the ideal case, we should experimentally follow the dynamics of mRNA numbers in the same cell over time in order to obtain an unbiased picture of gene expression regulation in cell cycle in unperturbed situations and during stress. This is currently technically very challenging. To overcome this limitation, we used static single cell data and performed an *in silico* synchronization.

This approach revealed detailed quantitative insights into transcriptional regulation and profiles with and without stress that were not available from studies with synchronized cells, since all available synchronization methods have adverse side effects and only limited duration of synchrony.

We found enduring basal levels of expression of all three genes, but highest for *SIC1*. Therefore, we considered phases of high and low expression in the computational model. The model, revealed—as expected—lower noise during periods of high transcription than during low transcription for each mRNA species. However, we found lower noise for *SIC1* than for *CLN2* or *CLB5* in both high and low expression periods.

A potential consequence of the basal levels of *SIC1* may be to prevent strong transcriptional bursts and, thus, higher transcript noise,^45,46^ as well as cell cycle timing noise.^43,47^ FRET measurements revealed that Sic1 binds to Clb2, Clb3, and Clb5 suggesting a role for Sic1 in different phases and not restricted to G1/S transition.^48^ Indeed, these cyclins lost their oscillation-like periodicities in cells lacking Sic1.^49^ As sole CKI of B-type cyclins, Sic1 is therefore important to act as cell cycle timer under normal conditions, but as brake under stress. We suggest that the basal transcription of *SIC1* might be an advantage to enable smoother and less burst-like expression peaks, as well as to provide basal Sic1 protein levels in case the cell cycle must suddenly be arrested.

### Single mRNA FISH and in silico synchronization revealed precise quantification and timing of gene expression

We observed a sharper peak of *SIC1* in late mitosis (Ana and T/C) dropping in early G1. In comparison, previous works using population assays reported that *SIC1* mRNA and Sic1 protein levels remain high until S phase entrance.^50,51^ This expanded expression of *SIC1* might be due to the limitations of synchronization to resolve the short phases of mitosis. In addition previous studies differed on the initiation of *SIC1* main transcription in or after anaphase.^12–14^ We could show that the *SIC1* peak in mitosis starts during anaphase.

*CLN2* and *CLB5* mRNA maxima in late G1 agree with previous reports for population studies using α-factor or elutriation for synchronization in G1 for *CLN2*^52,53^ and for *CLB5*,^8,35,54^ but timing of cell cycle phases differs between synchronized cultures and non-synchronized cultures, due to the shortened cell cycle length of chemically synchronized cells, which keep gaining size without cell cycle progression. *CLB5* mRNA was found to decay after exit from S phase leading to minimal levels of *CLB5* mRNA^55,56^ and Clb5 protein^20^ from S throughout M until G1. The *CLN2* maximum was 4-fold higher than that of *CLB5* as previously observed for mRNA^23,24^ and protein levels.^57^ This might be due to a positive feedback loop of Cln2 triggering its own expression.^52,58,59^

The unexpected transcription upshift in late mitosis of both cyclins might have been overlooked in previous studies due to the loss of synchrony at later cell cycle phases in populations synchronized in G1.^35^ A potential explanation for the mitotic transcription is that *CLN2* and *CLB5* transcription is mainly induced by the transcription factor complexes SBF (SCB-binding factor) and MBF (MCB-binding factor), respectively, in late G1.^10,11,60^ In late mitosis, nuclear Whi5 inhibits *CLN2* transcription by binding to SBF.^43,61^ It was shown that Zinc finger protein Rme1, which has maximum expression at M/G1, induces promoter activity at *CLN2* loci.^62^ However, the repressor Nrm1 is inhibiting MBF outside G1 phase^63^ but is degraded at the exit of mitosis.^64^ As a result, there may be a short time window allowing transcription of cyclins when SBF and MBF are bound to the promoters and not yet inhibited.

### Osmostress induces arrests in early G1, as well as in G2 and long-term effects

We placed special emphasis on the effect of stress—here osmotic stress—on gene expression. While most previous studies focused on the initial phase of adaptation using synchronization methods, we also investigated long-term effects of hyperosmolarity on cell cycle progression. Measuring the fraction of cells per cell cycle phase at different times of stress, we observed different effects on cell cycle progression throughout the single cell cycle phases. Even after 90 min the initial subdivision of the population into cell cycle phases was not fully restored, consistent with previously reported two hours for recovery.^65^ Cells accumulated in gap phases, early G1 and G2, with a preference for early G1. This may indicate that arrest in early G1 before START, which prevents DNA replication, may be more important than arrest in G2, which delays nuclear division and allows for DNA repair. Consequently, the fractions of cells decreased in late G1, S, and mitosis. Previous studies showed that S phase is delayed and elongated upon osmostress to ensure genomic integrity by preventing collisions between the replication and transcription machineries due to transcriptional bursts of stress-induced genes.^20,66^ We could not observe S phase elongation because, although some individual cells may have had longer S phases, the overall S phase group decreased as cells arrested in early G1. Since osmostress hits non-synchronized cells at arbitrary cell cycle phase and affects all phases, measuring osmoadaptation in single living cells using e.g., microfluidic devices, cell cycle markers and MS2 tagging system^67^ would provide deeper insight into the exact timing of cell cycle progression and gene expression under stress conditions.

Intriguingly, after osmostress we observed cells having two spindle pole bodies but no bud, exhibiting a loss of synchrony between DNA replication and budding. This fraction of cells reached a maximum after 30 min of stress. A comparable phenomenon was observed when osmostress was applied close to or after START.^22^ Adrover and colleagues suggested that after START cells lost the ability to arrest in G1 in response to stress and initiated DNA replication whereas budding was delayed by about 30 min.

We measured different inhibition duration and recovery patterns for *CLN2* and *CLB5* indicating distinct osmoregulation mechanisms of these cyclins. In unstressed cells, their transcription is triggered synchronously^11,68^ ensuring simultaneous budding and DNA replication. However, upon osmostress Hog1 appears to downregulate *CLN2* and *CLB5* transcription independently and for different periods. Whereas Hog1 is specifically recruited to the *CLB5* promoter inhibiting transcription,^22^ Hog1 targets Whi5 to downregulate *CLN2*.^69^

In contrast to the transient inhibition of *CLN2* and *CLB5* transcription, *SIC1* expression was less affected upon hyperosmolarity. The differential behavior of *SIC1* and the cyclins is reflected by their different roles in cell cycle progression and stress response. Sic1 as inhibitor of B-type cyclins and, hence, inhibitor of cell cycle progression may be crucial to control the delay of cell cycle events required under stress. In contrast, Cln2 and Clb5 are activators of the G1/S transition.

## Conclusion

Combination of single molecule FISH and microscopy with a mathematical model revealed detailed and quantitative regulatory patterns in gene expression of three well-known G1/S regulators. Detection of a ubiquitous basal expression level, an unexpected mitotic expression, the determination of molecular noise, as well as dynamics of transcription recovery upon hyperosmolarity required a quantitative single cell resolution method. Furthermore, the use of morphological cell cycle markers can avoid adverse side effects and the averaging effect of synchronized population assays. Our mathematical model enabled computational cell cycle synchronization and to rationalize the experimental data. Future investigations could address the question whether these patterns are specific to *SIC1*, *CLN2*, and *CLB5* or general to other cell cycle regulators in yeast or to homologous genes in other organisms.

## Materials and methods

### Strain and plasmid construction

For allocation of the cell cycle state of individual cells we cloned a marker strain, with mTurquoise labeled spindle pole bodies and TagGFP labeled Whi5.^41^ We used BY4741 (*MATa his3Δ1 leu2Δ0 met15Δ0 ura3Δ0*) haploid yeast strain as parental strain (EUROSCARF n. Y00000). For detailed protocol, see Supplementary Information Material and Methods.

### Yeast growth conditions

Cells were grown overnight at 30 °C in Yeast extract Peptone Dextrose (YPD) medium containing 2% glucose (w/v). The next morning cells were diluted to OD_600_ ~0.05, allowed to grow for several generations, OD_600_ was measured every hour for calculation of the cell cycle duration. When culture reached OD_600_ ~0.3 cells were used for all following protocols. For application of osmostress, a final concentration of 0.4 M sodium chloride was added. After different time point (15, 30, 45, 60 and 90 minutes) samples were taken and fixed with 4% (w/v) paraformaldehyde (PFA) for 45 min at room temperature.

### Single molecule RNA-Fluorescent in situ Hybridization (smFISH) procedure

For each transcript, *SIC1*, *CLN2*, and *CLB5*, a set of approximately 35 fluorescently labeled probes was used as described in ref.^70^ and ordered from Biosearch Technology (California, USA). The list of the probe sequences and features are in Supplementary Tables S3-S5. Used fluorophores: Quasar® 570 for *SIC1* and *CLN2* (excitation maxima: 548 nm, emission maxima: 566 nm), CAL Fluor Red® 610 for *CLB5* (excitation maxima: 590 nm, emission maxima: 610 nm).

The smFISH experiment was essentially performed as described in ref.^33^ and in ref.^71^ with two modifications: hybridization buffer contains 10%(v/v) formamide and hybridization was carried out for 4 h. For a more detailed protocol, see Supplementary Information Material and Methods.

### Microscopy image acquisition and analysis

Images were acquired with an Olympus IX81 epifluorescence microscope. For each experiment 10-25 images per time point were taken. For detailed information about microscopy and analysis, see Supplementary Information Material and Methods.

### Stochastic model of transcription regulation

The model as described in Table 1 has been stochastically simulated using the Gillespie algorithm. For simulation of mRNA time-courses, 2000 cells have been simulated. In order to have comparable values at comparable time points, snapshots of the system have been taken every minute, i.e., the current molecule numbers at every full minute have been recorded. The resulting lists of 129 values for one cell cycle have then been used to calculate means, standard deviations and coefficients of variation for each mRNA. However, the simulation started 15 min before cell cycle start (starting from anaphase of one cell cycle to the T/C phase of the next cell cycle). In case of osmotic stress, 250 min were simulated to cover 129 min of cell cycle, 15 min for anaphase and T/C phase, the delay due to osmostress and the up to 90 min of stress duration. The respective number of time points was analyzed, depending on the time point of stress. Code availability: The code can be sent upon request.

### Data availability statement

Experimental frequency distributions of mRNA numbers per cell of *SIC1*, *CLN2*, and *CLB5* under normal (Fig. 2b, c) and hyperosmotic (Fig. 5c, d) conditions are available as excel table in Supplementary Table S1. Numerical values of Fig. 5a are given in Fig. S6. Additional data that support the findings of this study are available from the corresponding author upon reasonable request.

## Electronic supplementary material

Suppliementary Material

Dataset 1

## Electronic supplementary material

**Supplementary information** accompanies the paper on the *npj Systems Biology and Applications* website (10.1038/s41540-018-0053-4).
