# Supplementary material for: Transcriptional timing and noise of yeast cell cycle regulators—a single cell and single molecule approach
Source: NPJ Syst Biol Appl. 2018 May 21;4:17. doi: 10.1038/s41540-018-0053-4 (PMC5962571; doi:10.1038/s41540-018-0053-4)
Supplement: Supplementary file 1 — Suppliementary Material [file 41540_2018_53_MOESM1_ESM.pdf]

Transcriptional timing and noise of yeast cell cycle regulators – a single cell and single molecule approach

Aouefa Amoussouvi[1,2], Lotte Teufel[1,2], Matthias Reis[1], Martin Seeger[1], Julia Katharina Schlichting[1],

Gabriele Schreiber[1], Andreas Herrmann[2], and Edda Klipp[1]

[1] Theoretical Biophysics, Institute of Biology, Humboldt-Universität zu Berlin

[2] Molecular Biophysics, Institute of Biology, Humboldt-Universität zu Berlin

Contact: Edda Klipp: edda.klipp@rz.hu-berlin.de

Supplementary Information

Content

1. Parameter estimation
2. Material and Methods
3. Supplementary Figures S1-S13
4. Supplementary Tables S1-S5
5. Biological replicates

1. Parameter estimation for transcriptional activity of *SIC1*, *CLN2*, and *CLB5* from the experimental mRNA distributions obtained by smFISH

1.1. Description of the model for parameter estimation

The basic structure of the model is shown in Fig. 1B. Considering that the number of mRNA molecules in the measurements is small (between 0 and 71 molecules per cell) and available in distribution form, we chose a stochastic modeling approach. For the same reason we were able to treat each gene separately. Consequently, the following reactions needed to be considered for parameter estimation:

$$\varnothing \xrightarrow{k(t)} \mathrm{mRNA}$$

$$\mathrm{mRNA} \xrightarrow{p} \varnothing$$

Transcription with a rate k_{1h} is started at a time $t_{S,0}$ and stopped (Signal S_i present, $k(t) = k_{1h}, \forall t_{S,0} \leq t \leq t_{S,e}$).
Since all promoters exhibited a basal activity (especially *SIC1*), at all other times of the cell cycle the genes were modeled to be transcribed with a rate k_{1l}(Signal S_i absent, $k(t) = k_{1l}, \forall t < t_{S,0}, t > t_{S,e}$). *CLN2* and *CLB5* have two transcription regions with rate k_{1h} labeled as “first” and “second”.

We implemented the stochastic part of the model using the exact solution of the master equation for monomolecular reaction networks (Jahnke and Huisinga, 2006). In our case, the reaction system only consists of one species, namely the number of mRNA molecules $n(t)$. The solution of the master equation is then a matrix $P(n, t|n_0, t_0)$ that describes the probability of a transition between n_0 molecules at a time t_0 and n molecules at time t. Please note that the definition of a monomolecular reaction according to (Jahnke and Huisinga, 2006, p. 4) includes only conversion, production from source or degradation, but neither (auto)catalytic nor splitting reactions. The form of the solution of the chemical master equation yielding this matrix depends on the type of initial conditions that will be discussed in the following.

Since our experiments do not reveal information about the past of a given cell, such as the number of molecules at the initial time, i.e. the start of the cell cycle, n_0 was assumed to be distributed according to a Poisson distribution rather than to take a certain value. This distribution was chosen because the measurements of n can be considered statistically independent of each other. In other words, the number of mRNA molecules $n(t)$ of a given cell does not depend on whether another cell with $n_0(t_0)$ molecules was measured before.

As already noted by (Gardiner, 2009, p. 269), proposition 2 in (Jahnke and Huisinga, 2006) states that the solution of the monomolecular master equation for a Poissonian initial distribution is a Poisson distribution. The parameter set of the latter distribution is given by the solution of the traditional reaction rate equations. For our system consisting of only one random variable $n(t)$ (the mRNA number) only one reaction rate equation, namely

$$\frac{d\langle n(t)\rangle}{dt} = k(t) - p \cdot \langle n(t)\rangle$$

Equation **1**

needs to be solved. $\langle n(t_0)\rangle$ is the initial value used for numerical solution of the equation that is also the parameter for the initial distribution. Note that the mentioned proposition also holds in the case of time dependent reaction rates such as our $k(t)$. Also consider that under the Poissonian initial condition

$$P(n_0, t_0) = \frac{\langle n(t_0)\rangle^{n(t_0)}}{n(t_0)!} e^{-\langle n(t_0)\rangle}$$

so our matrix $P(n, t|n_0, t_0)$ has identical row entries. This means that our solution takes a form that can be represented by a vector or one histogram (= mRNA distribution) per time point.

One last step is missing to simulate a histogram for parameter fitting as depicted in Supplementary Figure S4. The cells in our experiments are measured not at a certain point time point during the cell cycle but are assigned to a certain stage of the cell cycle using the described imaging approach (see Figure 1D and Section "Cell cycle phase markers"). Consequently we need to average the mRNA distributions for all time points between the start and the end of a given cell cycle phase. This finally allows for comparison of the simulated mRNA distributions with the experimental data for parameter estimation.

This semi-analytical approach enabled us to estimate the parameters $k_{1h,1l}$, $t_{S,0,S,e}$ and p of the model in about the same computation time an exclusively deterministic ordinary differential equation approach would take. The algorithm used for parameter estimation is described in the following Section SI 1.2.

1.2. Calculation of likelihood for parameters estimation

Since the experimental mRNA distributions are given in discrete histogram form and the number of cells in each bin of the histograms is small, a χ^2 test could not be applied (Baker and Cousins, 1984). The reason is that a χ^2 test, here in Neyman's form

$$\chi^2 = \sum_{i=1}^{N} \frac{(m_i - y_i)^2}{m_i}$$

where m_i is the number of events (cells) in histogram bin i and y_i the prediction of the model, amounts to the implicit assumption of a Gaussian error distribution.

That is, the conditional probability of observing m_i given $y_i$can be written as

$$\log L_{\text{Normal}} = \log \prod_{i=1}^{N} P(m_i|y_i) = -\frac{1}{2}\sum_{i=1}^{N}\left(\log(2\pi m_i) + 2\frac{(m_i - y_i)^2}{m_i}\right)$$

Equation **2**

where y_i is the mean and and $m_i/\sqrt{2}$ to be the variance of the Gaussian. Since the logarithm is a strictly increasing function, we can obtain the maximum of the likelihood in a numerically more convenient form by optimizing in the log domain. Note that the additive

term $-log(2\pi m_i)$ does not play a role when maximizing the likelihood. We now see that the minimization of χ^2 is equivalent to the maximization of L_{Normal} .

There are two intuitive reasons why the Gaussian distribution cannot describe the measurement statistics. Firstly, a Gaussian error distribution would assign a non-vanishing probability density to negative m_i, which is unphysical since we cannot count a negative number of cells. Secondly, the Gaussian distribution is a distribution describing continuous random variables, but in our case we are counting a discrete number of cells.

As an alternative approach a Poissonian error model was chosen under the assumption that the single cells in each bin were measured independently of each other. The likelihood of a histogram given a model y_i is therefore given by

$$L = \prod_{i=1}^{N} \frac{y_i^{m_i}}{m_i!} e^{-y_i}$$

Equation **3**

As described in Section SI 1.3 the model y_i was normalized to conserve the area, which is the total number of cells in the histogram, of the measurement. This guarantees that data bins that contain a low number of cells are considered to have a larger "measurement noise" than bins with a high number. To understand this, it is instructive to consider that for a Poisson distribution the variance is equal to the mean, so the standard deviation $\sqrt{y_i}$, here interpreted as "measurement noise", decreases comparably for larger mean y_i. Put in other words, the more cells we counted for a given bin i, the higher the information we have about the system is.

1.3. Description of the algorithm to calculate likelihood for parameters estimation

The objective function (likelihood) was calculated by the following algorithm:

1. Calculate $\langle n(t) \rangle$ by solving Equation 1 numerically.
2. Since the ODE-solver algorithm used to obtain $\langle n(t) \rangle$ returns the function at non-equidistant time points, these are interpolated to obtain m equally spaced samples. This is necessary because the integration over each cell cycle phase (step 4) would otherwise yield incorrect results. Consequently the cell cycle average distribution would depend on the sampling density defined by the ODE solver.
3. For each sampling point $t_i \in t_1, \ldots, t_M$ (with $t_0 = 0$ and $t_M = 129$ min), store a Poisson distribution with mean $\langle n(t_i) \rangle$.
4. For every cell cycle phase $j = 0, \ldots, 6$, starting at t_j and t_{j+1} (except $j = 6$), sum up all histograms between t_j and t_{j+1}.
5. Normalize each histogram to reach the same number of cells as in the experiments.
6. Calculate the likelihood function by Equation 3.

The described likelihood was then maximized with respect to the model parameters $k_{1h}, k_{1l}, p, t_{S,0}$ and $t_{S,e}$. Optimization was performed with a line search algorithm implemented in the R function optim() using a quasi-Newton method with box constraints (L-BFGS-B method).

2. Material and Methods

2.1. Cloning strategy

In order to clone plasmids with removable selection marker and fluorescent proteins as PCR template, pUG72 (Euroscarf accession number P30117) was used. TagGFP and mTurquoise respectively were PCR amplified and cloned in PstI site of pUG72 in order to generate plasmids containing ORFs for fluorescent proteins and Ura3 selection marker flanked by two loxP sites. Transformation cassettes for homologous integration were PCR amplified with primer pairs shown in Supplementary Table S2. Integration cassettes were transformed in BY4741 and transformed clones were selected on minimal medium agar-plates lacking uracil. Successful integration was controlled microscopically. The Ura3 selection marker was removed by expression of Cre recombinase from plasmid pSH68 (Euroscarf accession number P30674). We observed similar growth rates for the wild type and the Whi5-TagGFP/Spc42-mTurquoise double-tagged strain, indicating that the tagging did not affect cell growth and viability.

2.2. Single molecule RNA-Fluorescent *in situ* Hybridization (smFISH) procedure

Cells were spheroplasted in buffer B containing 100U/ml lyticase, 20mM ribonucleoside vanadyl complex (VRC) and 2% (v/v) β-mercaptoethanol for 15 min at 30°C. Afterwards cells were in buffer B and stored in 70% ethanol at -20°C. For the hybridization, cells were rehydrated in 2xSSC for 5 min at RT, then in 2xSSC, 10% formamide for 5 min at RT and finally incubated in hybridization solution made of 2xSSC, 10% formamide, 1mg/ml BSA, 10mM VRC, 0.5mg/ml Escherichia coli tRNA, 0.5mg salmon sperm DNA, 0.1 g/ml dextran sulfate, 5mM sodium phosphate pH 7.5 and 0.25µM labeled smFISH DNA probes for 4 hours at 37°C. After hybridization cells were washed three times in 2xSSC, 10%(v/v) formamide for 15 min at 37°C. Afterwards cells were incubated in 1xSSC containing 5ng/ml DAPI for 5 min at RT and washed in 1xSSC for 5 min at RT. Cells were resuspended in 1xSSC and sedimented for 1hour at 4°C on poly-L-lysin coated coverslides. Non-attached cells were washed off with 1xSSC. Finally cells were mounted in 20%Mowiol/80% Vectashield (Vectashield hardset antifade mounting medium, Vector Laboratory, USA) and dried for microscopy.

2.3. Microscopy image acquisition

Images were acquired with Olympus IX81 epifluorescence microscope using an UPlanApo 100X, 1.35 numerical aperture oil- immersion objective (Olympus). An HBO 100 watts light source was used for illumination with an U-MWNIBA filter for TagGFP, modified U-MNG2 filter for Quasar®570, U-MCFPHQ for mTurquoise, U-MNG2 filter for CAL Fluor Red® 610 and U-MWU2 filter for DAPI. All filters were purchased from Olympus. The exposure time were 500ms for TagGFP, 1200ms for Quasar®570, 250ms for mTurquoise, 1200ms for CAL Fluor Red® 610 and 100ms for DAPI. Vertical stacks of 21 images with a z step size of 0.25µm were acquired using a Clara E Interline camera (Andor) with a 6.45µm pixel size CCD. MetaMorph® (Molecular Devices) software platform was used for instrument control and image acquisition.

2.4. Microscopy image analysis

Three-dimensional fluorescence image stacks were reduced to two-dimensional images by intensity projection along the z-axis using FIJI. Stacks of bright field images were reduced to best focus projections. Image stacks of DAPI stained nucleus, of mTurquoise-labeled spindle pole body and of smFISH labeled mRNA were reduced to maximal intensity projections. Image stacks of TagGFP-labeled Whi5 were reduced to average intensity projections. Using a FIJI plugin obtained from Robert Singer, New York, masks of the cell localizations were created from the hand-made segmentation of the cells on the bright field images. Detection of the single fluorescent particles of mRNA and spindle pole bodies (visible thanks to its component protein Spc42 tagged with mTurquoise) was made on the z-projections with Localize, an IDL function obtained from Daniel Larson, Bethesda and described in[28, 67]. The mRNA and spindle pole bodies spot information and cell masks were coupled with a custom written Python script. In total between 400 and 1000 cells were analyzed per time point and gene (precise numbers in Supplementary Table S1) and at least 1500 cells per time point for the cell cycle length information from two to four independent replicated experiments (replicates s. Supplement Information 5: Biological Replicates) pooled together.

2.4.1. Quantitative analysis of mRNA fluorescent

We calculated for each experiment and each gene the median intensity of the detected fluorescent spots and normalized the intensity of each spot by this median intensity to extract the number of mRNA in each fluorescent spot. Spots with less than 50% of the median value were excluded and considered false positives. A spot with an intensity of 50% to 150% of the median value was considered one mRNA molecule. A spot with an intensity of 150 % to 250% was considered two mRNAs and so on.

2.4.2. Identification of transcription sites

Our algorithm identified transcription sites (TS), which were nuclear fluorescence mRNA spots containing at least three times the intensity of a single mRNA spot. Nuclear localization was examined by visual screening according to the DAPI stained nucleus images. The fraction of cells with transcription sites (%) was calculated as the ratio of the number of cells with transcription sites to the total number of cells in the experiment.

2.4.3. Cell cycle segmentation

For assignment of cells to specific cell cycle phases, we used the Whi5-TagGFP/Spc42-mTurquoise double-tagged strain. We acquired bright field and fluorescence microscopy images of a non-synchronized population of this reporter strain. Each single cell from the non-synchronized cell population was assigned to different cell cycle phases (early G1, late G1, S, G2, prometa-/meta-phase (P/M), anaphase (Ana) or telophase/cytokinesis (T/C)) according to the patterns of the genetic and morphological cell cycle markers. The markers were the presence and size of a bud, the morphology of the DAPI stained nucleus, the number and localization of spindle pole body visualized by mTurquoise-labeled Spc42, and finally the localization of TagGFP-labeled Whi5 (Trcek et al., 2011). Whi5 is recruited to the nucleus between late M and early G1 and is cytoplasmic during the rest of the cell cycle (Costanzo et al., 2004).

The number of spindle pole bodies was automatically analyzed as described in the section (2.4.) whereas the other markers were analyzed by visual screening. All information was

collected in an excel sheet, where cell cycle phase was sorted using customer defined rules. The fractions of cell population in each cell cycle phase were processed using a pivot table in excel. The duplication time of this reporter strain was about 129 min in YPD at 30 °C and the duration of each phase was proportional to the number of cells in each specific phase (Trcek et al., 2011).

SUPPLEMENTAL REFERENCES

Baker, S., and Cousins, R.D. (1984). Clarification of the use of CHI-square and likelihood functions in fits to histograms. Nucl. Instruments Methods Phys. Res. *221*, 437–442.

Gardiner, C. (2009). Stochastic Methods: A Handbook for the Natural and Social Sciences (Springer Berlin Heidelberg).

Jahnke, T., and Huisinga, W. (2006). Solving the chemical master equation for monomolecular reaction systems analytically. J. Math. Biol. *54*, 1–26.

Trcek, T., Larson, D.R., Moldon, A., Query, C.C. & Singer, R.H. (2011), Single-molecule mRNA decay measurements reveal promoter- regulated mRNA stability in yeast. *Cell* **147**, 1484-1497.

Costanzo, M. Nishikawa, J. L., Tang, X., Millman, J. S., Schub, O., Breitkreuz, K., Dewar, D., Rupes, I., Andrews, B., Tyers, M. (2004), CDK activity antagonizes Whi5, an inhibitor of G1/S transcription in yeast. *Cell* **117**, 899-913.

Ball, D. A., Adames, N. R., Reischmann, N., Barik, D., Franck, C. T., Tyson, J. J., Peccoud, J. (2013), Measurement and modeling of transcriptional noise in the cell cycle regulatory network. *Cell cycle* **12**, 3203-3218.

3. Supplementary figures

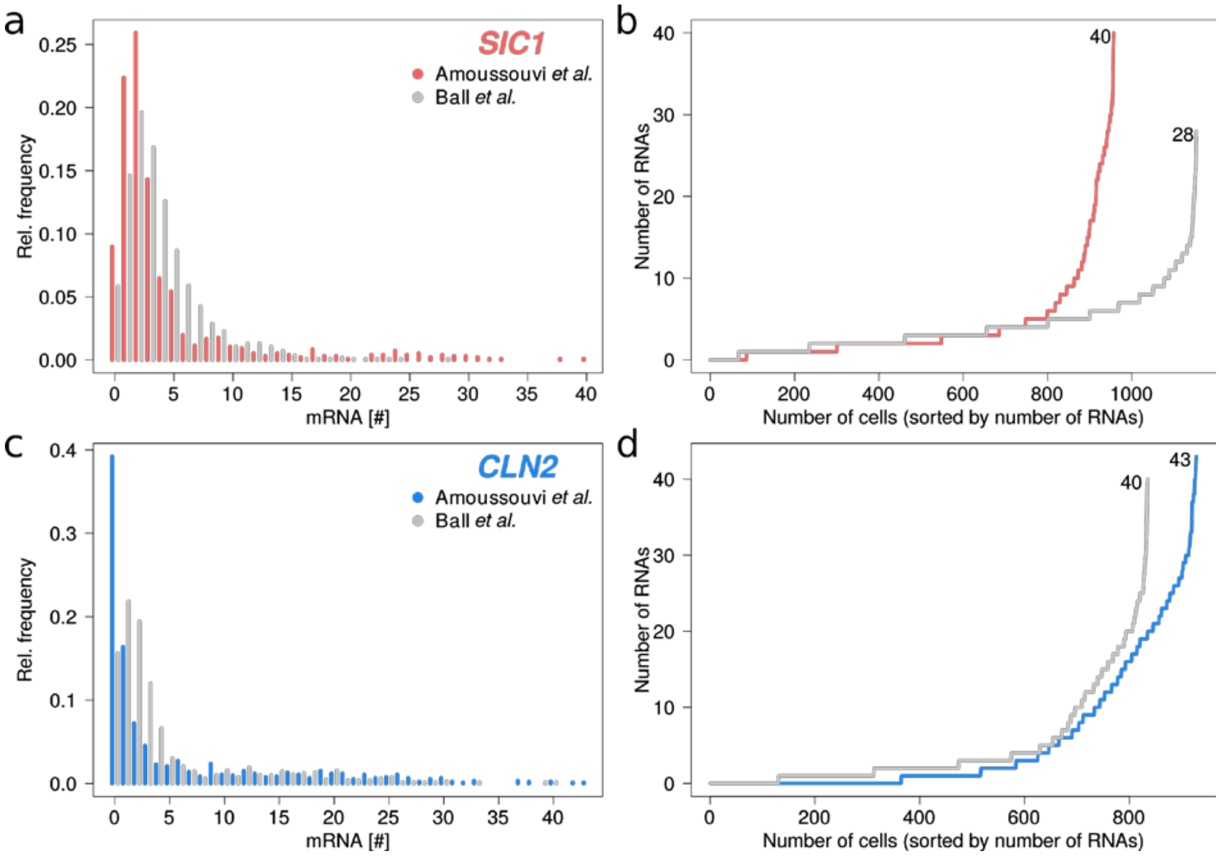

Supplementary Figure 1. Comparison of this study with Ball *et al.* (2013) for mRNA distributions of *SIC1* and *CLN2*. Comparisons for *SIC1* are given in (a)-(b) and for *CLN2* in (c)-(d). Data from this study are colored, whereas data from Ball *et al.* (2013) are gray. Data from this work correspond to number of detected fluorescent spots without normalization. (a) and (c) show histograms of relative frequencies of mRNA numbers over all cell cycle phases. (b) and (d) represent mRNA numbers per cell sorted by the number of mRNAs. The maximum number of mRNAs per cells is written close to the graphs. Means of mRNA per cell are in the same order of magnitude, i.e. for *SIC1* 4,11 in this study and 2,93 in Ball *et al.* as well as for *CLN2* 5,40 in this study and 4,24 in Ball *et al.* (discrepancies between single mRNA abundances can be partially attributed to systematic differences due to biological (yeast strain and culture media) and technical differences: we grew haploid BY4741 yeast strain in YPD while Ball and colleagues grew diploid BY4743 in SC medium).

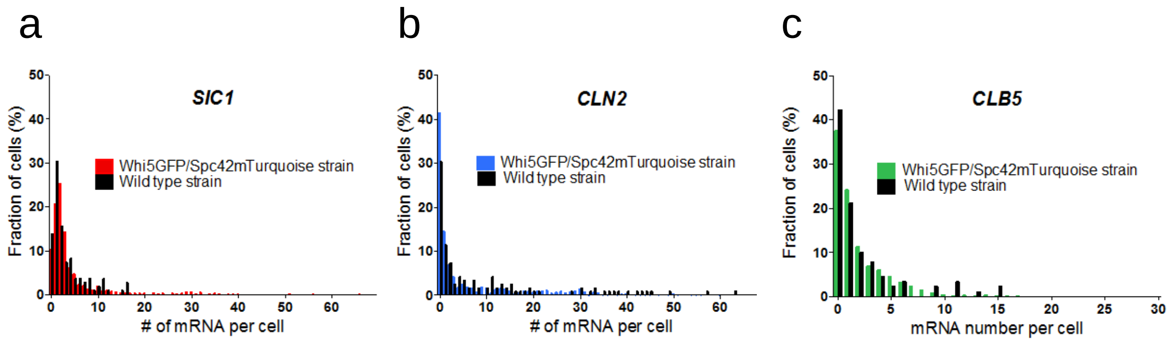

Supplementary Figure S2. Comparison of mRNA distributions of *SIC1*, *CLN2*, and *CLB5* in wild type and Whi5TagGFP/Spc42mTurquoise strains. Data of *SIC1*, *CLN2*, *CLB5* are shown in (a), (b) and (c), respectively. Data in wild type strain are shown in black and represent approximately 100 cells. Data in Whi5TagGFP/Spc42mTurquoise strain are shown in colors and represent approximately 900 cells.

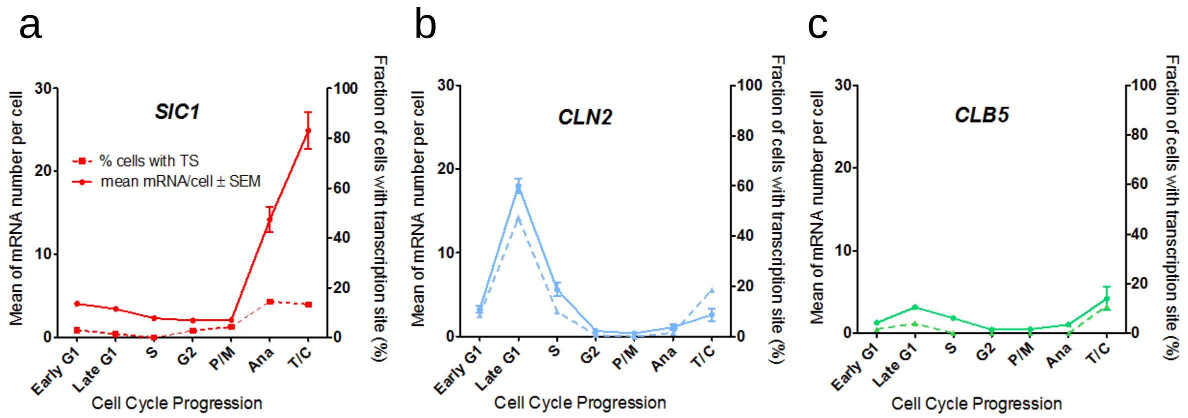

Supplementary Figure S3. Experimental mean mRNA numbers (± SEM, full lines) and fraction of cells with transcriptional sites (in %, dotted lines) in each cell cycle phase. Data for *SIC1*, *CLN2*, and *CLB5* are shown in (a), (b) and (c), respectively.

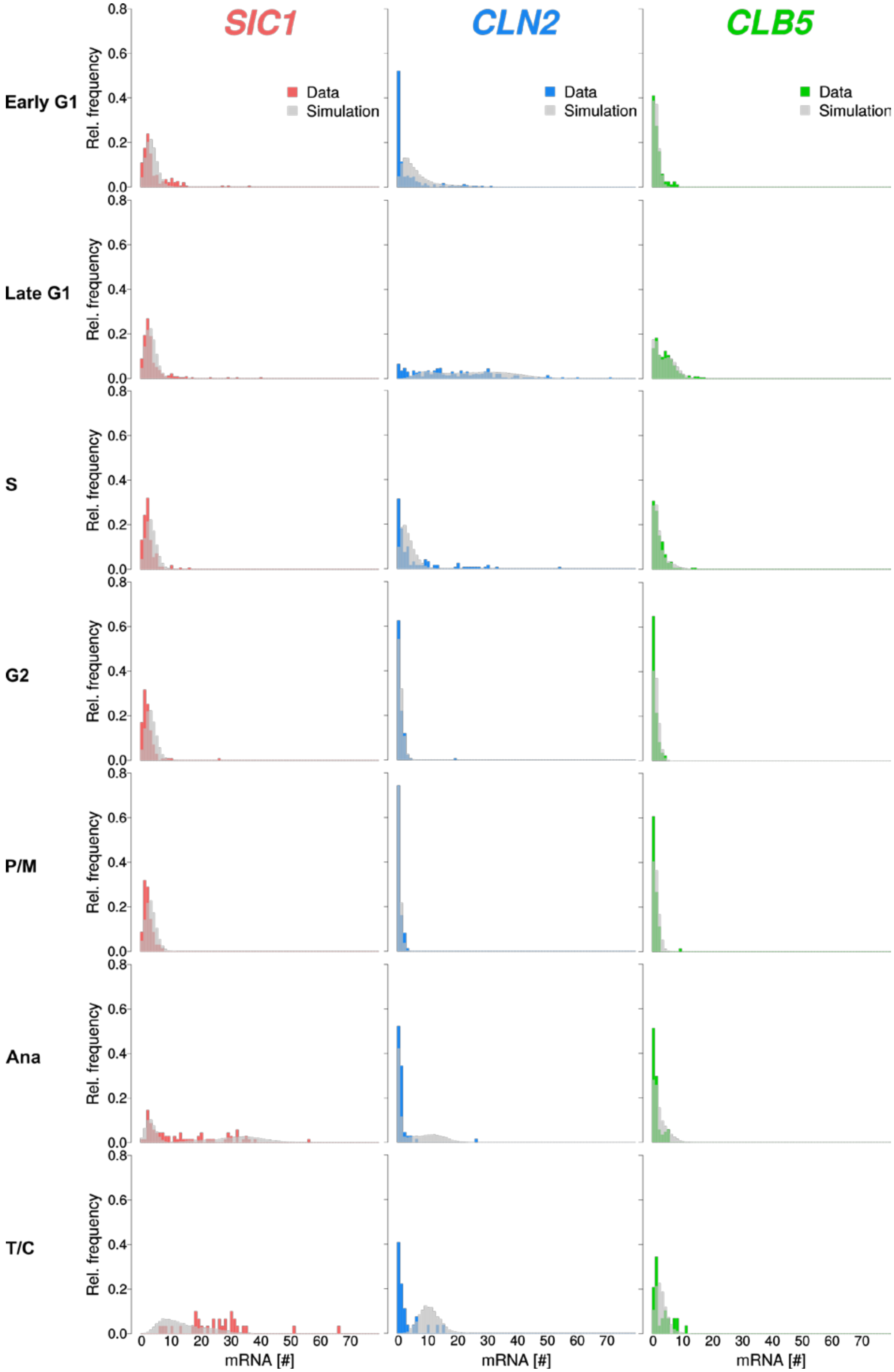
SIC1
CLN2
CLB5
Early G1
Late G1
S
G2
P/M
Ana
T/C
Rel. frequency
Data
Simulation
mRNA [#]
0.0
0.2
0.4
0.6
0.8
0 10 20 30 40 50 60 70

Supplementary Figure S4. Distributions of *SIC1*, *CLN2* and *CLB5* mRNA in each cell cycle phase. Data for *SIC1*, *CLN2* and *CLB5* are shown in left, middle and right columns, respectively. Histograms of relative frequency of mRNA numbers were obtained experimentally from smFISH assays (colors) and from computational simulations (gray).

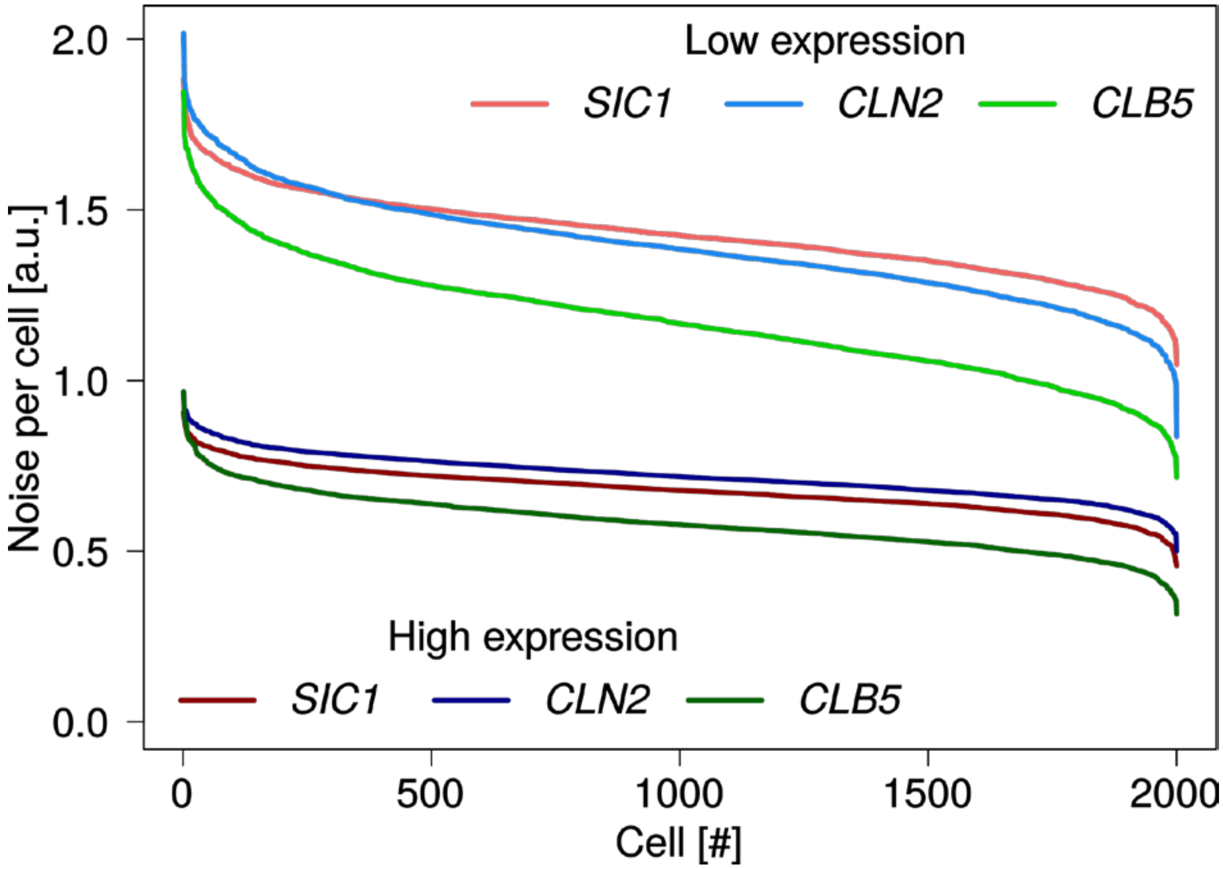

Supplementary Figure S5. Sorted noise of 2000 single cells measured for each cell individually over one cell cycle period. Comparison between high and low mRNA expression. Simulations were performed with the Gillespie algorithm using the equations and parameter values provided in Table 1. *SIC1* is shown in red, *CLN2* in blue, *CLB5* in green.

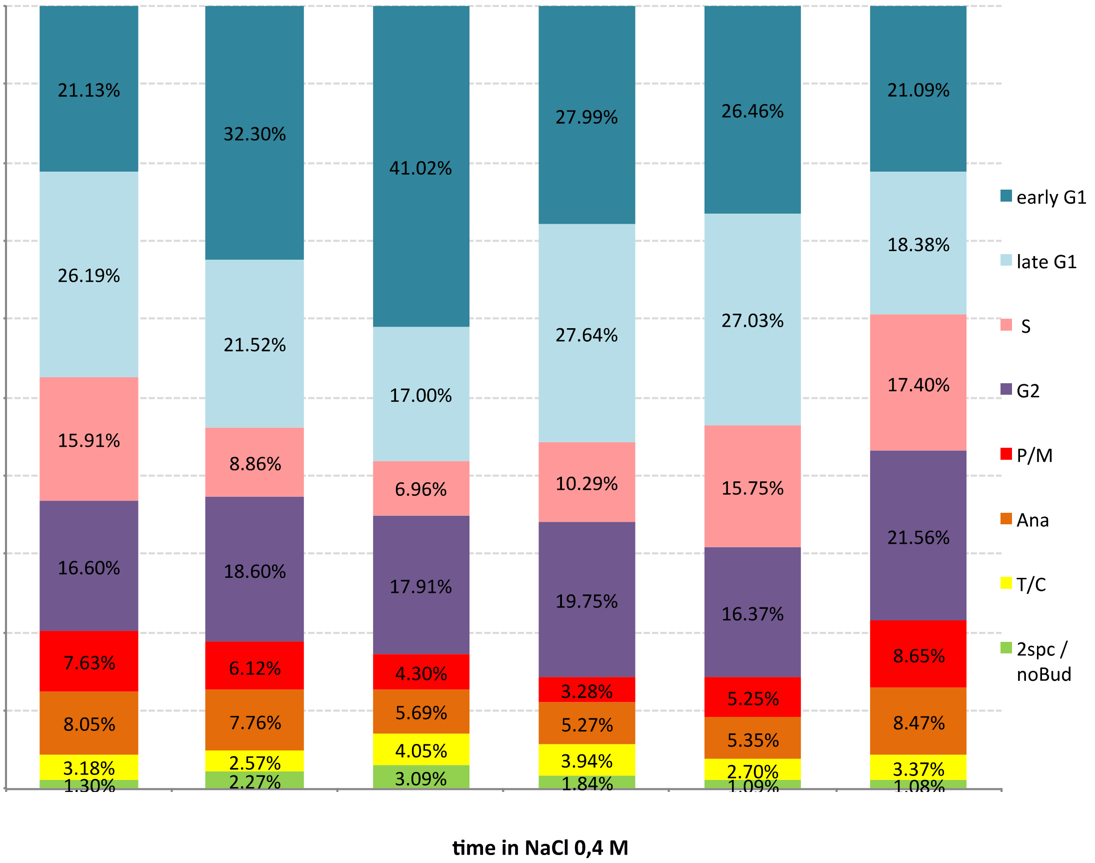

Supplementary Figure S6. Hyperosmolarity affects cell cycle progression. Quantitative distribution with percentage of cells in each cell cycle phase under normal growth condition (no stress) and under exposure to hyperosmolarity for 15, 30, 45, 60 and 90 min. Number of cell analyzed shown in Supplementary Table S6.

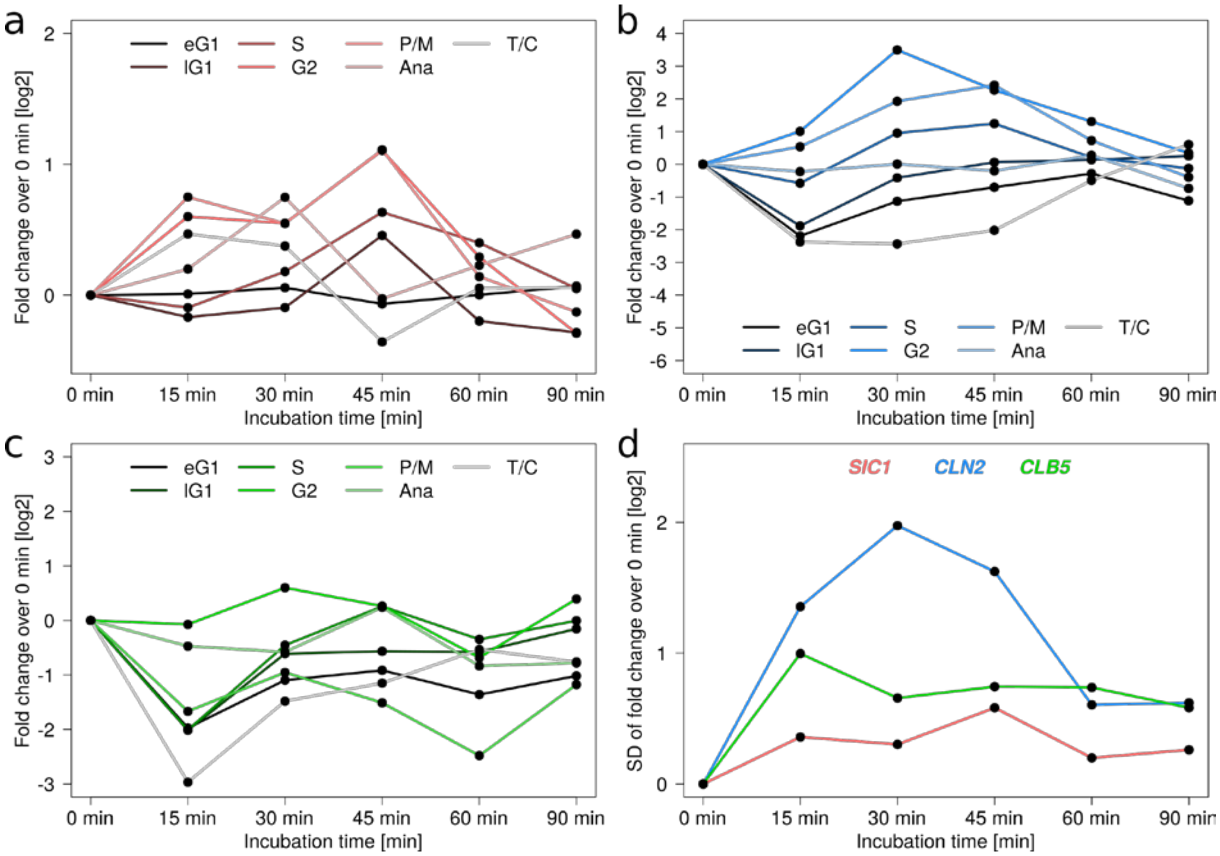

Supplementary Figure S7. Osmotic stress response presented as fold changes over no stress condition. (a), (b) and (c) show fold changes over 0 minutes osmotic stress (no stress condition) for *SIC1* (red), *CLN2* (blue) and *CLB5* (green), respectively. Fold changes are expressed in log_2 and calculated from the mean values per cell cycle phase. Fold changes are plotted for each stress time (0, 15, 30, 45, 60 and 90 minutes) and each cell cycle phase, whereby cell cycle phases are given as colored lines. (d) Standard deviation of fold changes per stress time and gene are depicted. Lines between data points in (a)-(d) are for visualization only.

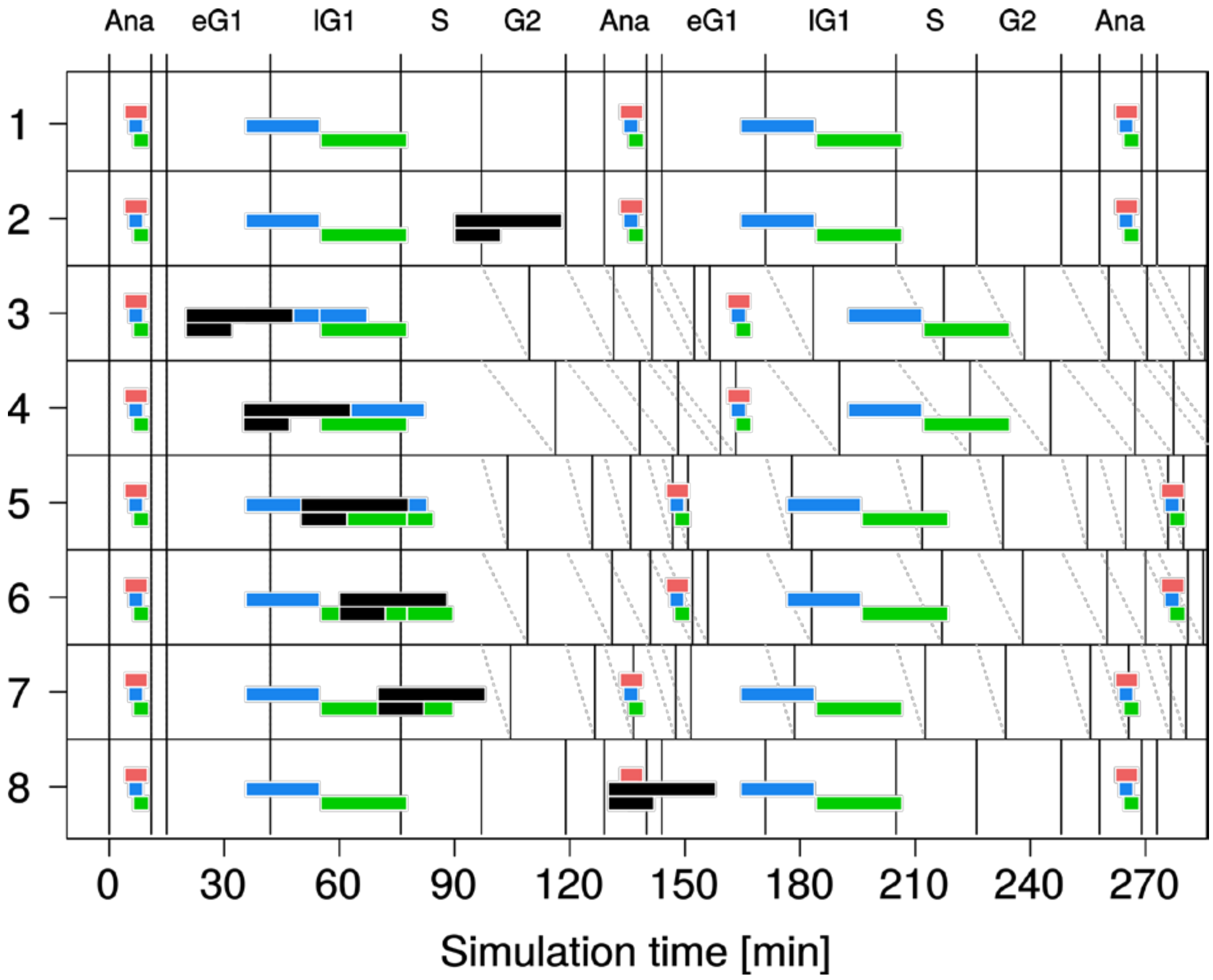

Supplementary Figure S8. Schematic of the temporal order of cell cycle phases and distinct active and inactive transcription periods of mRNA under osmostress. Active transcription periods for *SIC1*, *CLN2* and *CLB5* are represented by red, blue and green bars, respectively. The delay in transcriptional activity due to osmostress is depicted by black bars. The unnamed phases around Ana correspond to P/M and T/C. 1: No osmostress; no phase shifts. 2: Osmostress has no effect on high expression regions; no phase shifts. 3: Osmostress hits *CLN2* at the beginning of the high expression region; phases are shifted by the overlap between osmostress and the high transcription region of *CLN2.* 4. Osmostress overlaps the high transcription region of *CLN2*; phases are shifted by the stress duration of *CLN2.* 5. Osmostress cuts high transcription region of *CLN2* and hits *CLB5* at the beginning of its high transcription region; phases are shifted by the larger overlap between osmostress and the high transcription regions of *CLN2* or *CLB5*. 6. Osmostress is located insight high transcription region of *CLB5*; phases are shifted by the stress duration of *CLB5.* 7. Osmostress cuts high transcription region of *CLB5*; phases are shifted by the overlap between osmostress and the high transcription region of *CLB5*. 8. Osmostress has no effect on high transcription region of *SIC1* and second expression peaks of *CLN2* and *CLB5* aren't made up after being hit by osmostress; no phase shifts.

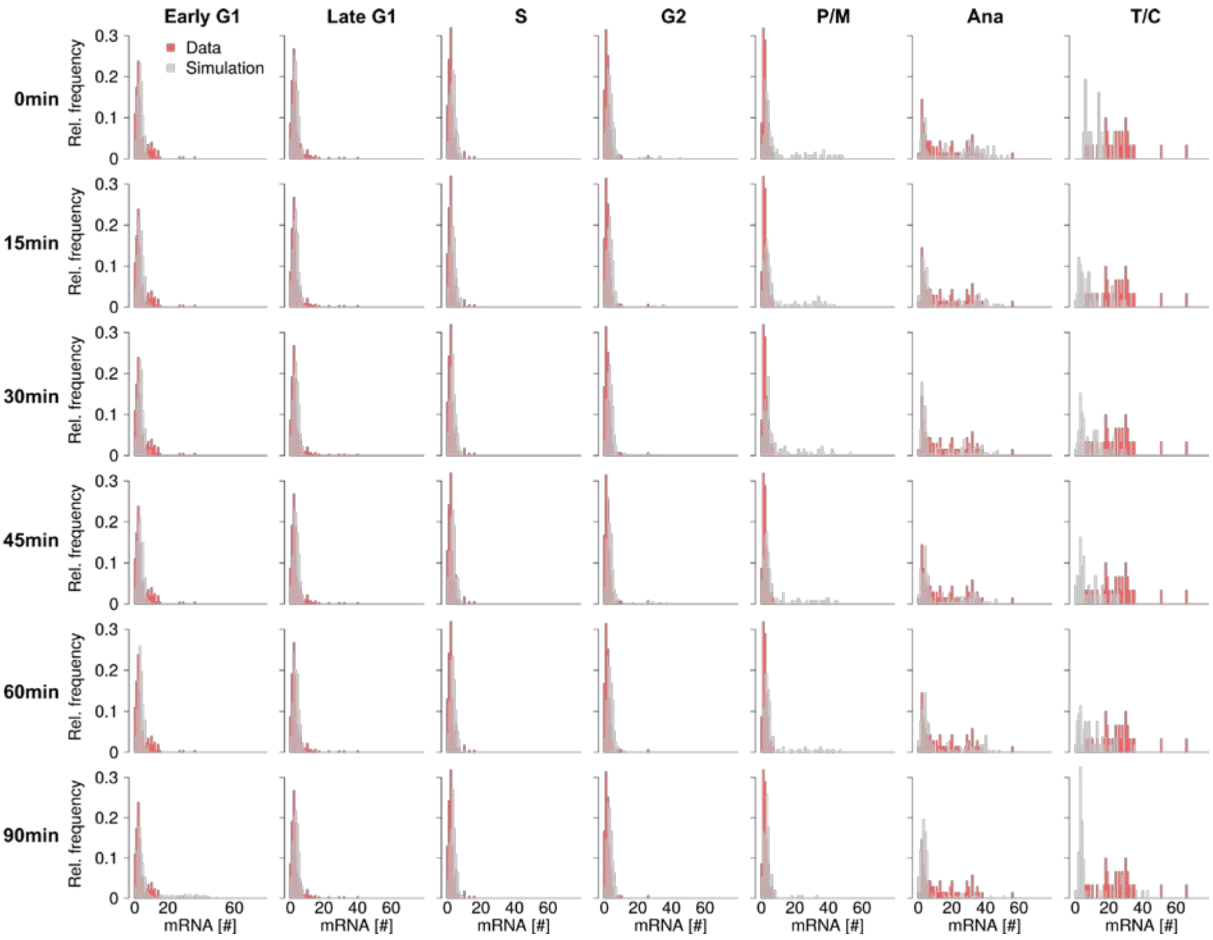

Supplementary Figure S9. Distributions of *SIC1* mRNA in each cell cycle phase under osmotic stress. Histograms of relative frequency of mRNA numbers were experimentally obtained from smFISH assays (red) and from computational simulations (gray). Osmotic stress was applied for 0 (no stress), 15, 30, 45, 60 and 90 min.

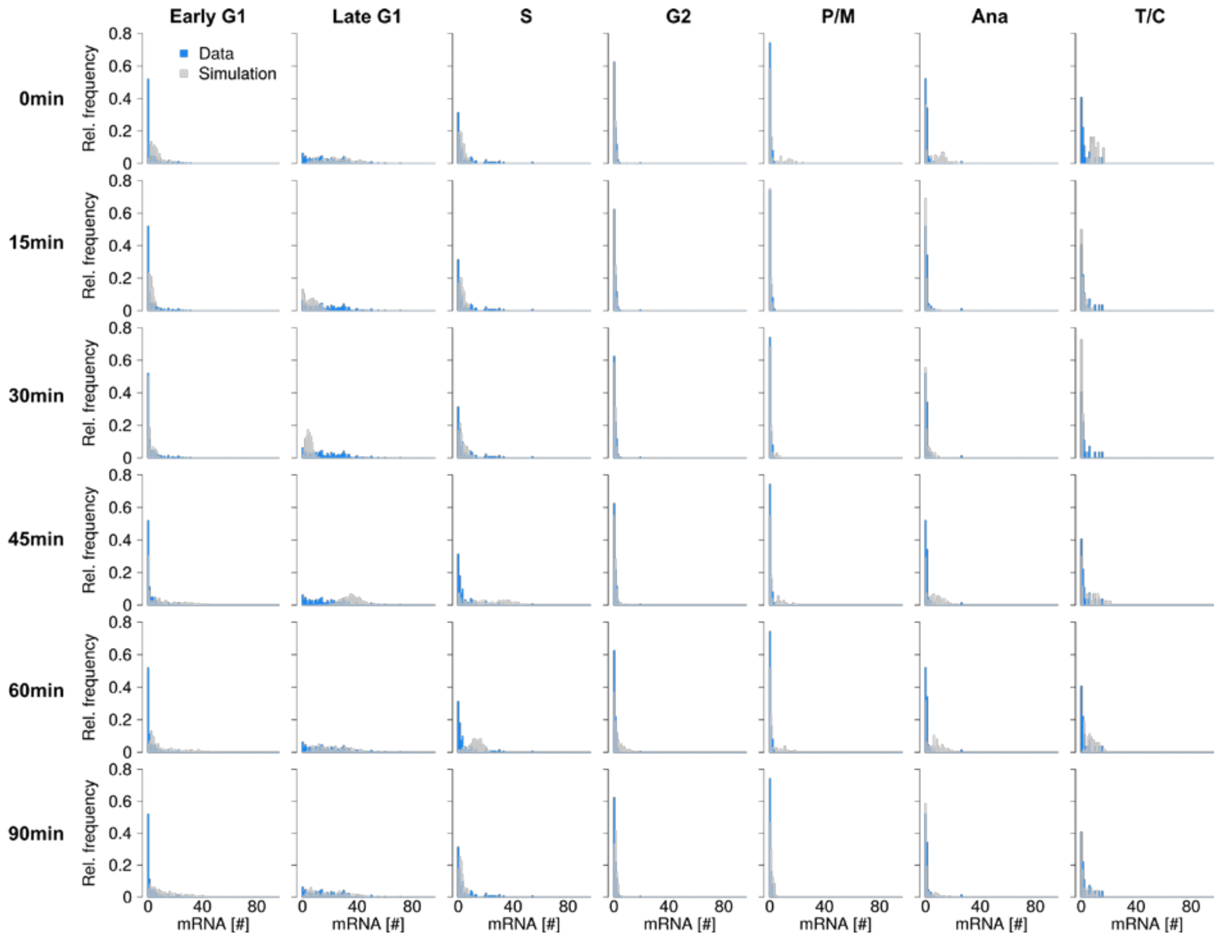

Supplementary Figure S10. Distributions of *CLN2* mRNA in each cell cycle phase under osmotic stress. Histograms of relative frequency of mRNA numbers were experimentally obtained from smFISH assays (blue) and from computational simulations (gray). Osmotic stress was applied for 0 (no stress), 15, 30, 45, 60 and 90 min.

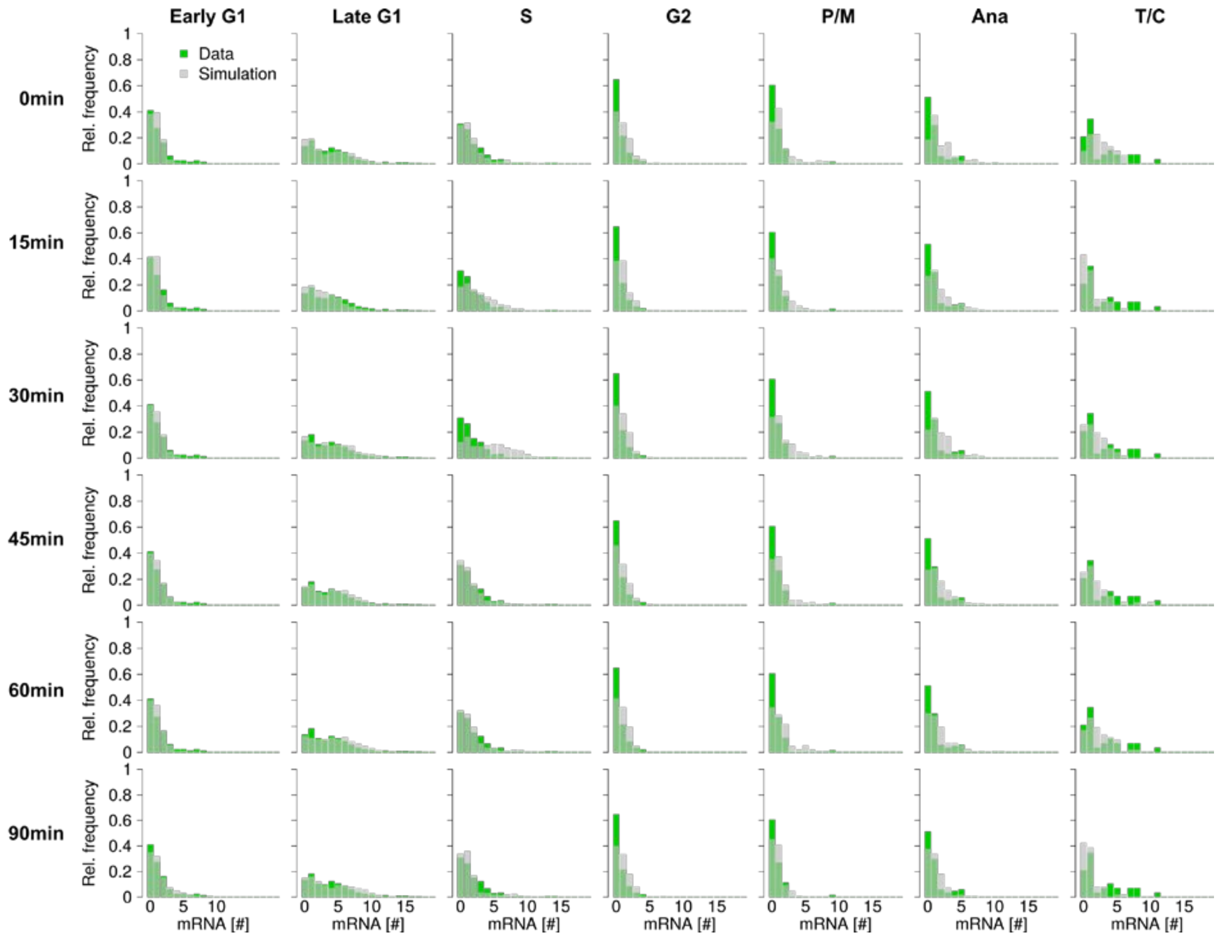

Supplementary Figure S11. Distributions of *CLB5* mRNA in each cell cycle phase under osmotic stress. Histograms of relative frequency of mRNA numbers were experimentally obtained from smFISH assays (green) and from computational simulations (gray). Osmotic stress was applied for 0 (no stress), 15, 30, 45, 60 and 90 min.

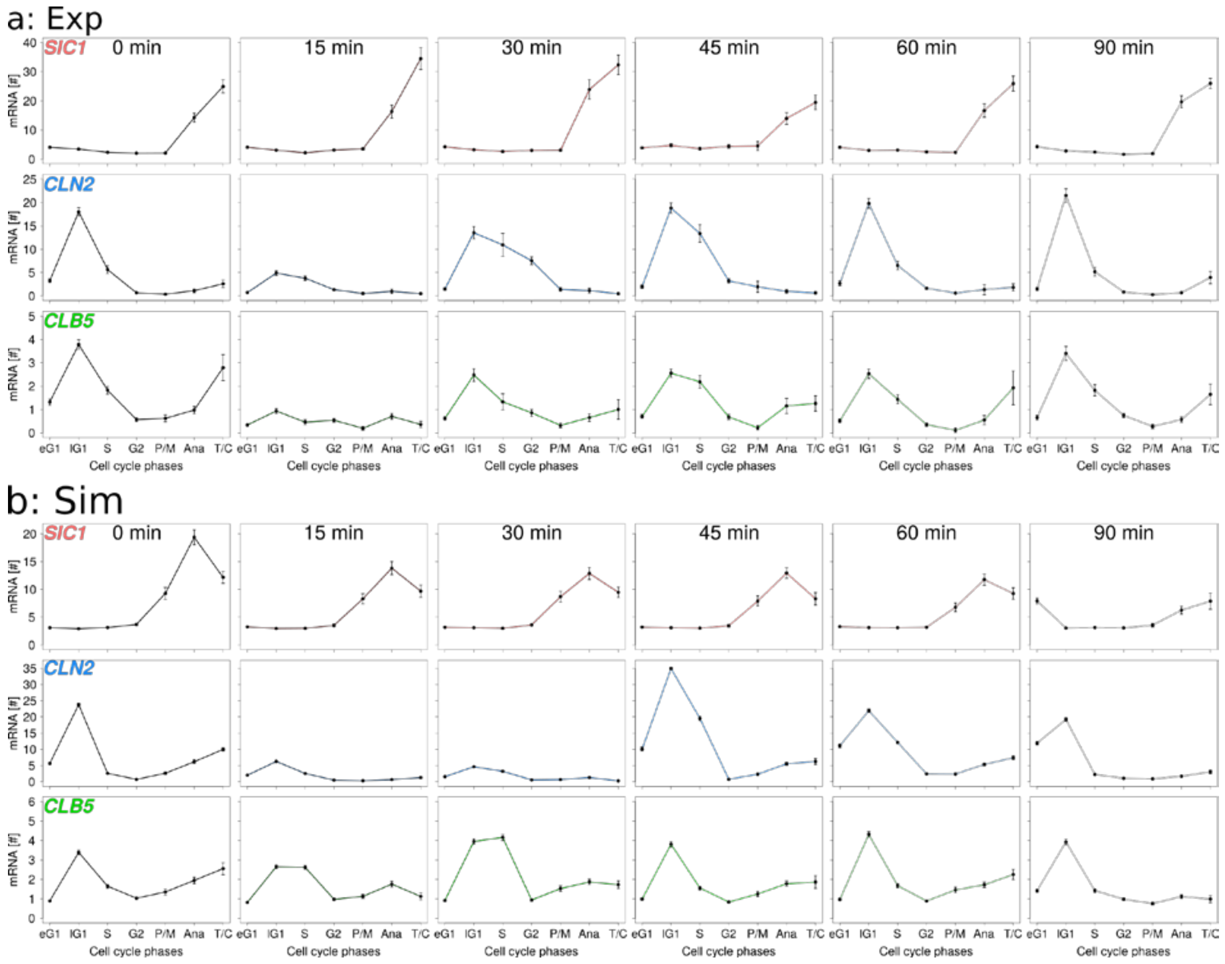

Supplementary Figure S12. Influence of osmostress on timing of mRNA abundaces for *SIC1*, *CLN2* and *CLB5*. Individual plots for experimental (a) and simulated (b) mRNA abundances per cell cycle phase shown in Figure 5d and e. Genes are presented in rows and stress times in columns. Data shown are mean ± SEM. Lines between data points in (a) and (b) are for visualization only.

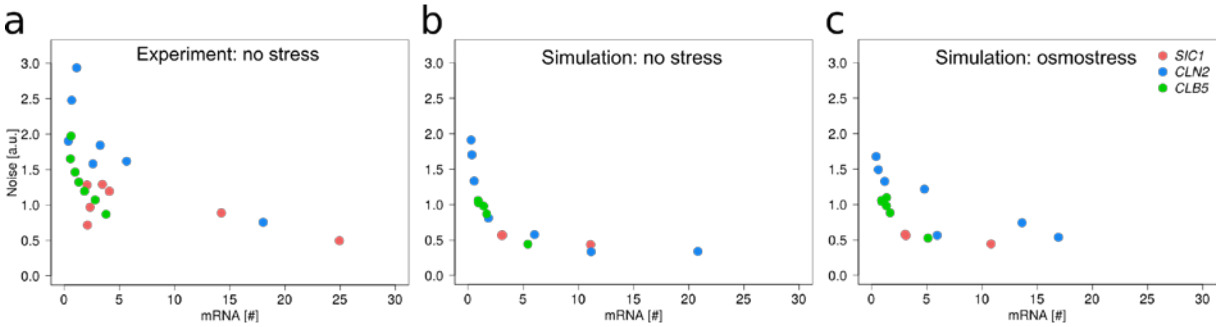

Supplementary Figure S13. Relationship between expression level and noise. In each diagram, the mean value of noise is plotted against the mean number of mRNAs for *SIC1*, *CLN2* and *CLB5* in each cell cycle phase. (a) Correlation coefficients for the experimental data under no stress condition are r_{SIC1} = -0.71, r_{CLN2} = -0.80, r_{CLB5} = -0.90 and r_{all} = -0.56, where r_{all} correspond to the correlation coefficient over all genes. (b) Simulations under no stress condition result in correlation coefficients r_{SIC1} = -1, r_{CLN2} = -0.78, r_{CLB5} = -0.99 and r_{all} = -0.62, and (c) correlation coefficients for simulations with osmostress are r_{SIC1} = -0.99, r_{CLN2} = -0.84, r_{CLB5} = -0.97 and r_{all} = -0.51. Correlation coefficients indicate strong correlations, means lower mRNA levels have higher noise levels. *SIC1* and *CLN2* show significantly different noise levels in the experiment ($p_{SIC1\text{-}CLN2}$ = 0.01, $p_{CLN2\text{-}CLB5}$ = 0.16, $p_{CLB5\text{-}SIC1}$ = 0.07), whereas *SIC1* and *CLB5* show significant differences in simulations with ($p_{SIC1\text{-}CLN2}$ = 0.20, $p_{CLN2\text{-}CLB5}$ = 0.90, $p_{CLB5\text{-}SIC1}$ = 0.02) and without osmostress ($p_{SIC1\text{-}CLN2}$ = 0.10, $p_{CLN2\text{-}CLB5}$ = 0.46, $p_{CLB5\text{-}SIC1}$ = 0.02). Only CLN2 has similar noise levels for the experiment and data under no stress condition (p_{SIC1} = 0.02, p_{CLN2} = 0.07, p_{CLB5} = 0.02). p values are calculated from with the Wilcoxon-Mann-Whitney test.

4. Supplementary Tables

Table S1: Experimental frequency distributions of mRNA numbers for *SIC1, CLB5* and *CLN2* per cell in each cell cycle phase.

See separate Excel sheet

Table S2. Properties of the oligonucleotides used for the cloning of the Whi5-TagGFP/Spc42-mTurquoise double tagged strain.

fw_Spc42_integ	5’-CTGAAAATAATATGTCAGAAACATTCGCAACTCCCACTCCCAATAATCGAGGAGCAGGT GCTGGTGCT-3’
rev_Spc42_integ	5’-GCTTTAAGAATGCGCCATACTCCTTAACTGCTTTTTAAATCATCAATTAGAAAAACTCAT CGAGCATC-3’
fw_Whi5_integ	5’-ACGGACGAAACGGAGCCCGAGTCGGATACCGAAGTGGAGACGTCTGCAGGAATGAG CGG-3’
rev_Whi5_integ	5’-CACTAACTCCGAGATTGCGGAGAAAAAACTCGTACTACCACAGCATAGGCCACTAGTG GATCTG-3’

Table S3. Properties of the smFISH oligonucleotide set for *SIC1*. Start and end positions are relative to the ORF of the gene.

Index	Name Probe	Sequence (5' to 3')	Start Position	End Position	Length (nucleotides)
1	Sic1_yeast_570_1	TTGGTGGGGTGGAAGGAGTC	3	22	20
2	Sic1_yeast_570_2	GCTGCGCAAGGTACCTAGTC	33	52	20
3	Sic1_yeast_570_3	TGCATTAGGGCACTAGAAC	68	86	19
4	Sic1_yeast_570_4	TAGGTTCTGTGAAGGCTTTT	104	123	20
5	Sic1_yeast_570_5	AGTTGTTGAGGGAGTGACAG	128	147	20
6	Sic1_yeast_570_6	TGCTAATAATGGCGCATTTT	158	177	20
7	Sic1_yeast_570_7	AGAGGTCATACCCATGTTCG	188	207	20
8	Sic1_yeast_570_8	GAGCGTTGAGGAGACGTAAG	220	239	20
9	Sic1_yeast_570_9	CACTGAAGATTTTGGAAACGG	241	261	21
10	Sic1_yeast_570_10	TCAAATTGGAATAGTGTCCTC	264	284	21
11	Sic1_yeast_570_11	ACTGTTCCATTATCATGACTT	285	305	21
12	Sic1_yeast_570_12	TGGTTCCTGCTCTTCCCT	307	324	18
13	Sic1_yeast_570_13	ATCCTATTTACACGACCCAA	325	344	20
14	Sic1_yeast_570_14	CACATTTTGCTGCGTGGGAA	350	369	20
15	Sic1_yeast_570_15	CTTCTTCTGCTGCATCTATA	372	391	20
16	Sic1_yeast_570_16	AGATGTAGGTCTGCTGGGG	417	435	19
17	Sic1_yeast_570_17	AGTGATAAATGTAACTGCCT	439	458	20
18	Sic1_yeast_570_18	TGTCTGATCAAACTCATCTCT	463	483	21
19	Sic1_yeast_570_19	GGCGTACCAGGTACATCTTT	502	521	20
20	Sic1_yeast_570_20	AATGTTATCACCTTGTCGCT	523	542	20
21	Sic1_yeast_570_21	CACTTTCTTGACTCCTGGCG	585	604	20
22	Sic1_yeast_570_22	CACCCGCACTGGATTGATGA	623	642	20
23	Sic1_yeast_570_23	TTCCTGAGTGACCAGTTCAT	665	684	20
24	Sic1_yeast_570_24	AACATTGCCCTTTTACGTTC	694	713	20
25	Sic1_yeast_570_25	ACGTCTTCTATATCTGGGTT	721	740	20
26	Sic1_yeast_570_26	CCCTTCTTATTGACATATGTTAT	742	764	23
27	Sic1_yeast_570_27	TCATCCGTTAACCTTCGTTT	778	797	20
28	Sic1_yeast_570_28	GGCTTGAATCTTCTCTTTTCT	798	818	21
29	Sic1_yeast_570_29	CAATGCTCTTGATCCCTAG	836	854	19

Table S4. Properties of the smFISH oligonucleotide set for *CLN2*. Start and end positions are relative to the ORF of the gene.

Index	Name Probe	Sequence (5' to 3')	Start Position	End Position	Length (nucleotides)
1	Cln2_yeast_570_1	CTTGGTTCAGCACTAGCCAT	1	20	20
2	Cln2_yeast_570_2	TAGCATTGATGACGAGTCCC	30	49	20
3	Cln2_yeast_570_3	CTGCATTAGATAGCTCAATCGG	64	85	22
4	Cln2_yeast_570_4	GGTGGTATTCTTGCAGCATT	105	124	20
5	Cln2_yeast_570_5	CTGCTGGTCTATTAGTTTTGGAT	173	195	23
6	Cln2_yeast_570_6	CTTGTTTCCACGGGGTTCAT	202	221	20
7	Cln2_yeast_570_7	GACCACAGACAGCTCGAACA	242	261	20
8	Cln2_yeast_570_8	CTCTTGGAACAATAGCGGTC	307	326	20
9	Cln2_yeast_570_9	ATTTGGCTTGGTCCCGTAAC	333	352	20
10	Cln2_yeast_570_10	TTAGCAGCCAACCAGAGACA	367	386	20
11	Cln2_yeast_570_11	GTGATTACAACCGCCCCAAG	389	408	20
12	Cln2_yeast_570_12	ATCTTCCGCCAGTAGGGATG	426	445	20
13	Cln2_yeast_570_13	GAGAGTCGAGGTATACGTGC	466	485	20
14	Cln2_yeast_570_14	AAGACCTGACCATCACCACA	502	521	20
15	Cln2_yeast_570_15	AGTTCGTATTGCATCAAACAATT	625	647	23
16	Cln2_yeast_570_16	GGGATAATTGAGACTGACGTT	689	709	21
17	Cln2_yeast_570_17	CCTCTCGTCTACAGTGGCAT	719	738	20
18	Cln2_yeast_570_18	TGCCACGCGGATACATCAAT	814	833	20
19	Cln2_yeast_570_19	GAATATGCCGTGCGATACTT	863	882	20
20	Cln2_yeast_570_20	CCGTGGTCTTGATTGGTGAA	898	917	20
21	Cln2_yeast_570_21	GCTTTCTGATGTCATTGGAGT	931	951	21
22	Cln2_yeast_570_22	ATGCCGTTCATTAAGGTACT	970	989	20
23	Cln2_yeast_570_23	ACACTTCCATCAAGGAGTTAGG	1000	1021	22
24	Cln2_yeast_570_24	GTAGAACACCATTGACCGTTT	1025	1045	21
25	Cln2_yeast_570_25	ATGTTCAAGTTGGATGCAATT	1098	1118	21
26	Cln2_yeast_570_26	AAAGAGCATGATGGGGTTGA	1138	1157	20
27	Cln2_yeast_570_27	TGAGGAAGCGGGCGAAGGA	1182	1200	19
28	Cln2_yeast_570_28	ATTGGAGTGTGGCTTTGAGA	1201	1220	20
29	Cln2_yeast_570_29	ATATTCCGGCTGAAAACGCT	1249	1268	20
30	Cln2_yeast_570_30	ACATACTTGGAGTGATTGGTGA	1279	1300	22
31	Cln2_yeast_570_31	CATATACTGTTTGACTGCTGCT	1313	1334	22
32	Cln2_yeast_570_32	ACCAGACTATTCACACTAACGG	1343	1364	22
33	Cln2_yeast_570_33	TGTTCGTAGATCCTTTGTTTGT	1376	1397	22
34	Cln2_yeast_570_34	ATTGCTGTTAGGACCCGTGA	1400	1419	20
35	Cln2_yeast_570_35	TGGTTTTCCTTGTTAGACTCATT	1456	1478	23
36	Cln2_yeast_570_36	ATTGAGGTAATGCGCCGTT	1488	1506	19
37	Cln2_yeast_570_37	AATGAAGCTTGTCTTGGGT	1515	1533	19
38	Cln2_yeast_570_38	CAGTTGGCGAGGGGAACATT	1542	1561	20
39	Cln2_yeast_570_39	AGGCACTGCTAGATTTACCG	1575	1594	20

Table S5. Properties of the smFISH oligonucleotide set for *CLB5*. Start and end positions are relative to the ORF of the gene.

Index	Name Probe	Sequence (5' to 3')	Start Position	End Position	Length (nucleotides)
1	Clb5_yeast_570_1	CTGCTCATGGTCGTGGTTCT	8	27	20
2	Clb5_yeast_570_2	CACAACTGCCTCTCATTTTCAT	56	77	22
3	Clb5_yeast_570_3	ATCCTGAACCTGCTGCTTAC	137	156	20
4	Clb5_yeast_570_4	CTGTTAAAGCCCTTCTTGGT	162	181	20
5	Clb5_yeast_570_5	CTCTTGTTCTGGCTTAAAGGAT	200	221	22
6	Clb5_yeast_570_6	CTTCGTACTTTGGCCGCCTT	238	257	20
7	Clb5_yeast_570_7	ACGGCGCTAACAATAGGTC	272	290	19
8	Clb5_yeast_570_8	TGCTCTGCTGCCGTTCGAT	314	332	19
9	Clb5_yeast_570_9	TGTTCACTATCGAAGCAGCAT	368	388	21
10	Clb5_yeast_570_10	ACTCACTCCTTCAGCGTCTA	401	420	20
11	Clb5_yeast_570_11	TCTAGGTCCTGCCAGCCTAC	427	446	20
12	Clb5_yeast_570_12	GCTACCATTGCAGTATCATCTTT	457	479	23
13	Clb5_yeast_570_13	TTGTGCGATGGTAACGTTTCT	525	545	21
14	Clb5_yeast_570_14	ACTTGGACGTTTTGTCGAGT	552	571	20
15	Clb5_yeast_570_15	GTTCTCATGGAAGGCCTCAA	577	596	20
16	Clb5_yeast_570_16	GCACCTCTACCAGCCAATCC	606	625	20
17	Clb5_yeast_570_17	ACGTTTCCGGATAGCATTGA	636	655	20
18	Clb5_yeast_570_18	TGCCGCGATGAAAAGTGAGG	737	756	20
19	Clb5_yeast_570_19	CGCGCCGTCAGTGATATAAG	797	816	20
20	Clb5_yeast_570_20	TGAGCATGAACATTTCCGCA	840	859	20
21	Clb5_yeast_570_21	CCTTAGGAAATTGAGTGGGTT	889	909	21
22	Clb5_yeast_570_22	ATCATCTGCCTTGGAGATCCT	910	930	21
23	Clb5_yeast_570_23	GGTGGCAGCAGTAGGCAT	974	991	18
24	Clb5_yeast_570_24	GCCATTGCGCTTACGGTAGA	1012	1031	20
25	Clb5_yeast_570_25	CTGTTGGTCATTCTTCTCGC	1042	1061	20
26	Clb5_yeast_570_26	ATGCTGTAGTGTTCCATTCCA	1078	1098	21
27	Clb5_yeast_570_27	ATTGGATCGATACCACCACT	1102	1121	20
28	Clb5_yeast_570_28	ATGCAGAGAGACTGAAACGC	1132	1151	20
29	Clb5_yeast_570_29	GTTTTGGAACTAGCGATGTCTTT	1162	1184	23
30	Clb5_yeast_570_30	TGGAAATAAACAGAGCCATACCT	1222	1244	23
31	Clb5_yeast_570_31	GCATTTCGGATGTACACCACT	1253	1273	21

5. Biological Replicates

For each gene and time point up to four experiments were performed. Distributions were reproducible and experiments were merged together for analysis. Supplementary Table S6 shows total number of cells analyzed per cell cycle phase and time point under optimal growth conditions (no stress) and under osmotic stress. Furthermore plots of mean mRNA distributions for each cell cycle phase (left) and percentage of cells containing transcription sites (right) per phase are shown. Below each plot a table including number of cells per cell cycle phase (left) and number of cells containing transcription sites (right) are depicted.

Supplementary Table S6. Total number of cells analyzed per cell cycle phase and time point under optimal growth conditions (no stress) and under osmotic stress. These numbers served to generate cell cycle phase durations under optimal growth conditions (no stress) (Figure 1d) and under osmotic stress (Figure 5a and Supplementary Figure S6).

	early G1	late G1	S	G2	P/M	Ana	T/C	2spc / noBud	Total # of cells (=all phases)
no stress	551	683	415	433	199	210	83	34	2608
15 min	554	369	152	319	105	133	44	39	1715
30 min	678	281	115	296	71	94	67	51	1653
45 min	547	540	201	386	64	103	77	36	1954
60 min	509	520	303	315	101	103	52	21	1924
90 min	451	393	372	461	185	181	72	23	2138
